# Conversion of Medium-Sized Lactams to α-Vinyl or α-Acetylenyl Azacycles via *N*,*O*-Acetal TMS Ethers

**DOI:** 10.3390/molecules23113023

**Published:** 2018-11-19

**Authors:** Minjun Kim, Jaebong Jang, Goyoung Choi, Sungkyun Chung, Changjin Lim, Joonseong Hur, Hyun Su Kim, Younghwa Na, Woo Sung Son, Young-Ger Suh, Jong-Wha Jung, Seok-Ho Kim

**Affiliations:** 1 College of Pharmacy, Research Institute of Pharmaceutical Sciences, Kyungpook National University, Daegu 41566, Korea; mymkmj2@naver.com (M.K.); photoin7492@naver.com (G.C.); 2 Department of Cancer Biology, Dana-Faber Cancer Institute, Boston, MA 02215, USA; jaebong.jang@gmail.com; 3 Department of Biological Chemistry and Molecular Pharmacology, Harvard Medical School, Boston, MA 02215, USA; 4 Department of Pharmacy, College of Pharmacy and Institute of Pharmaceutical Sciences, CHA University, 120 Haeryong-ro, Pocheon 11160, Korea; wjdtjdrbs123@naver.com (S.C.); koryoi0709@gmail.com (C.L.); khs8812@snu.ac.kr (H.S.K.); yna7315@cha.ac.kr (Y.N.); wson@cha.ac.kr (W.S.S.); ygsuh@cha.ac.kr (Y.-G.S.); 5 College of Pharmacy, Seoul National University, 1 Gwanak-ro, Gwanak-gu, Seoul 08826, Korea; hjs1120@snu.ac.kr

**Keywords:** medium-sized lactam, amidoalkylation, α-vinyl or α-acetylenyl azacycles

## Abstract

α-Vinyl or α-acetylenyl azacycles were easily synthesized from 7- to 9-membered lactams and 6- to 9-membered lactams via *N*,*O*-acetal trimethylsilyl (TMS) ethers. Organocopper and organostannane reagents afforded reasonable yields for the respective *N*-acyliminium ion vinylation and acetylenylation intermediates generated from *N*,*O*-acetal TMS ethers in the presence of a Lewis acid.

## 1. Introduction

The conversion of lactams to their corresponding azacycles is an efficient route for the preparation of functionalized azacycles. It involves amide bond manipulations, however, the nucleophilic addition to amides is limited due to the poor electrophilicity of amide carbonyls. Therefore, α-amidoalkylation of reactive iminium ions derived from amides is an effective method for providing azacycles with scaffold diversity [1,2]. Many 5- and 6-membered lactams and azacycles exist in Nature, and the conversion of these lactams via α-amidoalkylation has been extensively studied. For instance, approaches involving thioamides [3,4,5], sulfonyl amides [6], *N*-alkoxylactams [7], formamidines [8], enamine triflates/phosphates [9,10] and *N*,*O*-acetals [11,12,13] have been developed for conversion to α-vinyl and α-acetylenyl azacycles. Moreover, these methods have been used for the preparation of key intermediates for the assembly of structurally diverse azacycles, ranging from a simple bicyclic to complex polycyclic systems [1] in combination with various synthetic methods including metathesis [6] and aza-Claisen rearrangements [14]. Recent developments in organometallic chemistry also have enabled concise and efficient synthesis of α-vinyl and α-acetylenyl azacycles from cycloalkylamines as surrogates for lactams via direct C–H activation [15] and photoredox reactions [16]. However, most of the reported methods have been limited to the synthesis of 5- and 6-membered azacycles. In contrast, the conversion of medium- to macrocyclic lactams to their corresponding azacycles has not been extensively investigated. Thus, a lack of concise and efficient methods for such conversions has been a major challenge in the synthesis of azacycles in which the high ring strain energy might give rise to undesirable ring openings. Conventional methods generate unstable intermediates from medium- to macrocyclic lactams, resulting in imines or enamines which would be readily hydrolyzed to the corresponding amino aldehyde [17]. In addition, a series of synthetic steps were required for the preparation of medium-sized 2-vinylazacycles even after extensive attempts to apply the reported methods [14]. In this regard, there is growing demand for efficient methods to convert medium-sized lactams to their corresponding azacycles with α-vinyl or α-acetylenyl functionality. Herein, we report a novel synthetic method for the conversion of medium-sized lactam rings to α-vinyl and α-acetylenyl azacycles using *N*,*O*-acetal trimethylsilyl (TMS) ethers as *N*-acyliminium ion precursors.

**Scheme 1:**
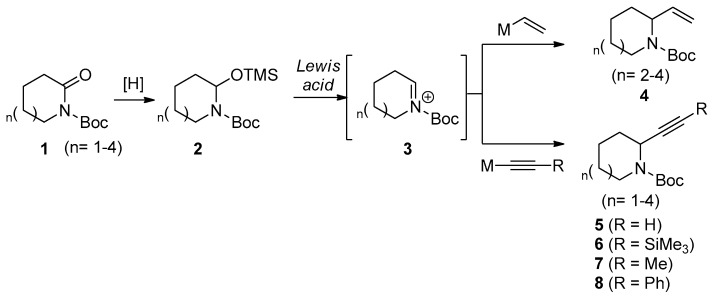
Conversion of medium-sized lactams to α-vinyl and α-acetylenyl azacycles via *N*,*O*-acetal TMS ethers.

The conversion of lactams to α-substituted azacycles involves the partial reduction and addition of a given nucleophile. The order of the two reactions is likely an important factor. For the conversion of medium-sized lactams, the introduction of a vinyl or acetylene nucleophile after the partial reduction of the lactam is likely to be more effective than the reverse sequence because of ring opening at the hemiaminal intermediate of medium-sized lactam. Considering the stability of the hemiaminal intermediate, *N*,*O*-acetal TMS ethers have been shown to be excellent precursors for reactions with medium- to macrocyclic azacycles that proceed via intermediate *N*-acyliminium ions [17]. Thus, we expected that α-vinyl and α-acetylenyl azacycles **4** and **5** could be prepared using *N*,*O*-acetal TMS ether **2** with proper nucleophiles via *N*-acyliminium ions **3** in the presence of a Lewis acid, as shown in Scheme 1.

## 2. Results and Discussion

### 2.1. Preparation of N,O-Acetal TMS Ether

**Scheme 2:**
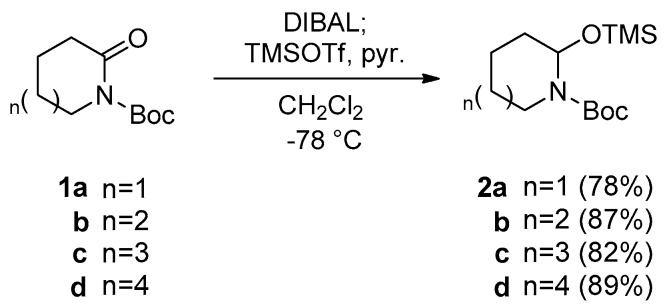
Preparation of *N*,*O*-acetal TMS ethers [16].

Our work commenced with the preparation of *N*,*O*-acetal TMS ethers. The requisite *N*,*O*-acetal TMS ethers were prepared from 6- to 9-membered lactams by using previously reported procedures [18], as shown in Scheme 2. In short, protected lactams **1a**–**d** were reduced with DIBAL, and trapping of the resulting *N*,*O*-acetal with trimethylsilyl triflate (TMSOTf) afforded the desired *N*,*O*-acetal TMS ethers **2a**–**d** in high yields.

### 2.2. Optimization of α-Vinylation

The vinylation of the 9-membered *N*,*O*-acetal TMS ether **2d** was performed using various vinyl metals as nucleophiles. As shown in Table 1, reaction of **2d** with the vinylcopper reagent derived from vinylmagnesium bromide and CuBr·SMe_2_ in the presence of BF_3_ OEt_2_ afforded the desired product **4d**, while the use of vinyllithium or vinylsilane resulted in a mixture of side products. The yield was considerably higher in tetrahydrofuran than in diethyl ether solvent. Other copper salts such as CuI and CuOTf could not be used instead of CuBr·SMe_2_. The coordinating characteristics of the anion that accompanies the Cu(I) ion might be attributed to the reaction. Under the same conditions, the vinylcopper reagent was reacted with *N*,*O*-acetal TMS ethers of the 7- and 8-membered azacycles **2b** and **2c** affording the α-vinylated adducts **4b** and **4c**, respectively. The *N*,*O*-acetal TMS ether **2a** did not undergo vinylation with the vinylcopper reagent. Previously, Neipp and Martin reported that an organocopper reagent derived from vinylmagnesium bromide could not produce a vinyl adduct from the 6-membered cyclic precursor of the *N*-acyliminium ion [6], whereas Collado et al. reported the highly efficient addition of Grignard-derived organocopper reagents to 5-membered cyclic *N*,*O*-acetals. 

### 2.3. Optimization α-Acetylenylation

Next, various acetylene nucleophiles with *N*,*O*-acetal TMS ether **2d** were reacted to afford α-acetylenyl azacycle **5d**. As shown in Table 2, the organocopper, organomagnesium, and organolithium reagents yielded unsuccessful results (entries 1–3, respectively). Although the use of trimethylsilyl cyanide (TMSCN) as a nucleophile for the reaction of *N*,*O*-acetal TMS ethers to afford α-cyanated cycloalkylamines is reported by Suh et al. [17], the use of trimethylsilylacetylene could not provide the desired α-acetylenyl azacycle **5d** (Entry 4). After extensive efforts, an organotin reagent was successful for the synthesis of α-acetylenyl azacycle **5d** from *N*,*O*-acetal TMS ether **2d** in the presence of a Lewis acid. Various solvents including dichloromethane, toluene, and diethyl ether except tetrahydrofuran (entries 5–8) were tolerable for the reaction. It was reported that tetrahydrofuran was not suitable for some reactions of *N*,*O*-acetal TMS ethers [19], even though tetrahydrofuran was the best solvent for vinylation of *N*,*O*-acetal TMS ethers in Table 1. BF_3_·OEt_2_ and TMSOTf were effective Lewis acids for the generation of the *N*-acyliminium ion, which readily reacted with the organotin reagent to afford the desired adduct **5d** (entries 5,11). The treatment of *N*,*O*-acetal TMS ether **2d** with the organotin reagent in the presence of SnCl_4_ or TiCl_4_ afforded a mixture of unidentified and possibly decomposed side products.

After optimizing the reaction conditions, we explored the scope of the reaction by using various substituted acetylenes. The required stannylacetylene reagents were purchased from commercial vendors or prepared as previously reported [20]. As shown in Table 3, *N*,*O*-acetal TMS ether **2** derived from 6- to 9-membered lactams **1** were easily reacted with various substituted stannylacetylenes to afford the corresponding α-acetylenyl azacycles **5**–**8** in reasonable yields. 

Various substituents including hydrogen, alkylsilane, alkyl, and aryl groups were well tolerated. For the 7- to 9-membered cyclic *N*,*O*-acetal TMS ethers, stannylacetylenes with electron donating groups, including Me or Ph, efficiently provided the desired α-acetylenyl azacycles in high yields. The chemical yield was notably lower in reactions for the 6-membered azacycles (entries 1–4) compared to the others. Enamine formation might be attributed to the unexpected low yield in those cases, and which was supported by the isolation of *N*-Boc-1,2,3,4-tetrahydropyridine as a major side product. Spectral data of the isolated *N*-Boc-1,2,3,4-tetrahydropyridine was identical to the reported data [21].

## 3. Materials and Methods

### 3.1. General Information

Unless noted otherwise, all starting materials and solvents were used as obtained from commercial suppliers without further purification. Organic solvents used in this study were dried over appropriate drying agents and distilled prior to use. Thin layer chromatography was carried out using Merck silica gel 60 F_254_ plates (Merck, Kenilworth, NJ, USA), and flash chromatography was performed automatically with Isolera (Biotage, Uppsala, Sweden) or manually using Merck silica gel 60 (0.040–0.063 mm, 230–400 mesh, Merck, Kenilworth, NJ, USA). ^1^H- and ^13^C-NMR spectra were recorded using JEOL-500 (JEOL, Tokyo, Japan) and AVANCE-500 (Brucker, Billerica, MA, USA) spectrometers. ^1^H- and ^13^C-NMR chemical shifts are reported in parts per million (ppm) relative to TMS, with the residual solvent peak used as an internal reference. ^1^H-NMR data were reported in the order of chemical shift, multiplicity (br, broad signal; s, singlet; d, doublet; t, triplet; q, quartet; quint, quintet; m, multiplet and/or multiple resonances), number of protons, and coupling constant in Hertz (Hz). Infrared spectra were recorded on Cary 630 FT-IR spectrometer (Agilent, Santa Clara, CA, USA). Low- and high-resolution mass spectra were obtained with JMS-700 (JEOL, Tokyo, Japan) and Q TOF 6530 (Agilent, Santa Clara, CA, USA) instruments. 

### 3.2. Synthesis

#### 3.2.1. Representative Procedure: Synthesis of *tert*-*Butyl 2-vinylazonane-1-carboxylate* (**4d**)

To a solution of CuBr·Me_2_S (308 mg, 1.5 mmol) in THF (4 mL) was added vinylmagnesium bromide (1.0 M solution in THF, 1.5 mL) at −50 °C and the mixture was slowly warmed to −40 °C for 30 min. Then, the reaction mixture was cooled to –78 °C and neat BF_3_·OEt_2_ (185 μL, 1.5 mmol) was added. After being kept at −78 °C for 5 mins, *N*,*O*-acetal TMS ether **2d** (95 mg, 0.3 mmol) in THF (5 mL) was transferred via cannula into the reaction mixture. The mixture was stirred at −78 °C for 3 h and then allowed to warm to room temperature over 2 h. The reaction was diluted with saturated aqueous NH_4_Cl (10 mL) and was extracted with Et_2_O. The organic extracts were dried over MgSO_4_ and concentrated under reduced pressure. The residue was purified by column chromatography on silica gel (EtOAc:*n*-hexane = 1:20) to afford the desired product **4d** (48 mg, 0.19 mmol). Yield 63%, colorless oil; ^1^H-NMR (CDCl_3_) *δ* 5.71 (m, 1H), 5.05–4.93 (m, 2H), 4.57 and 4.36 (m, 1H), 3.47 (m, 1H), 2.79 (m, 1H), 1.85 (m, 2H), 1.83–1.48 (m, 19H); ^13^C-NMR (CDCl_3_) *δ* 139.08, 138.96, 114.18, 79.30, 77.60, 31.95, 30.34, 29.72, 29.38, 28.58, 27.36, 27.05, 26.97, 24.25, 24.05, 22.71, 14.14; FT-IR (thin film, neat) ν_max_ 2924, 1692, 1455, 1403 cm^−1^; HRMS (ESI+) found 198.1489 [calculated for C_11_H_20_NO_2_ ([M + H − C_4_H_8_]^+^): 198.1488].

The following compounds were similarly prepared (Supplementary Materials):

*tert-Butyl 2-vinylazepane-1-carboxylate* (**4b**) Yield 65%, colorless oil; ^1^H-NMR (CDCl_3_) *δ* 5.71 (m, 1H), 5.01–4.95 (m, 2H), 4.59 and 4.34 (m, 1H), 3.86 and 3.66 (m, 1H), 2.65 (dd, 1H, *J* = 14.1, 11.4 Hz), 2.04 (m, 1H), 1.79–1.20 (m, 7H), 1.45 and 1.36 (s, 9H); ^13^C-NMR (CDCl_3_) *δ* 155.91, 138.60, 138.31, 112.84, 112.81, 79.18, 79.11, 57.88, 56.54, 42.33, 41.79, 33.94, 33.59, 29.74, 29.71, 29.25, 29.14, 28.56, 28.50, 25.27, 24.88; FT-IR (thin film, neat) ν_max_ 2927, 1692, 1408 cm^−1^; HRMS (ESI+) found 170.1176 [calculated for C_9_H_16_NO_2_ ([M + H − C_4_H_8_]^+^): 170.1174].

*tert-Butyl 2-vinylazocane-1-carboxylate* (**4c**) Yield 60%, colorless oil; ^1^H-NMR (500 MHz, CDCl_3_) *δ* 5.70–5.67 (m, 1H), 5.02–4.93 (m, 2H), 4.61 and 4.37 (m, 1H), 3.57–3.26 (m, 1H), 2.82 (m, 1H) 2.04–1.41 (m, 19H); ^13^C NMR (125 MHz, CDCl_3_) *δ* 155.98, 139.00, 138.77, 138.72, 138.58, 114.45, 114.29, 113.39, 79.18, 79.12, 78.99, 78.82, 58.27, 56.96, 41.92, 33.95, 30.56, 29.74, 29.27, 28.65, 28.62, 28.54, 28.05, 27.90, 27.16, 26.85, 26.77, 26.72, 26.48, 26.34, 26.22, 24.76, 24.44, 24.33; FT-IR (thin film, neat) ν_max_ 2928, 1693, 1475, 1403 cm^−1^; MS (EI+) *m*/*z* 254 [M + H]^+^; HRMS (FAB+) found 240.1968 [calculated for C_14_H_25_NO_2_ ([M + H]^+^): 240.1964].

#### 3.2.2. Representative Procedure: Synthesis of *tert-Butyl 2-ethynylazonane-1-carboxylate* (**5d**) 

To a stirred solution of *N*,*O*-acetal TMS ether **2d** (63 mg, 0.2 mmol) in CH_2_Cl_2_ (2 mL) was added ethynyltributylstannane (126 mg, 0.4 mmol) and BF_3_·OEt_2_ (25 μL, 0.2 mmol) at −78 °C and stirred for additional 30 min. The reaction mixture was slowly warmed to r.t. and Et_3_N was added to quench the reaction. The reaction mixture was filtered off insoluble precipitate and purified by flash silica gel column chromatography (EtOAc:*n*-hexane = 1:20) to afford the desired product **5d** (29 mg, 0.115 mmol). Yield 58%, a colorless oil; ^1^H-NMR (CDCl_3_) *δ* 5.05 and 4.79 (m, 1H), 3.57 and 3.51 (m, 1H), 3.07 (m, 1H), 2.23 (m, 1H), 1.95–1.25 (m, 12H), 1.48 (s, 9H); ^13^C-NMR (CDCl_3_) *δ* 156.14, 155.10, 83.44, 83.25, 80.01, 79.92, 70.88, 70.79, 48.98, 47.93, 44.73, 43.92, 32.47, 31.58, 29.71, 28.53, 21.17, 26.62, 26.45, 25.20, 25.09, 24.92, 24.67, 24.60, 24.51; IR (ATR-IR) ν_max_ 3300, 2919, 1687, 1399, 1162 cm^−1^; HRMS (ESI+) found 196.1336 [calculated C_11_H_18_NO_2_ ([M + H − C_4_H_8_]^+^): 196.1332].

The following compounds were similarly prepared (Supplementary Materials):

*tert-Butyl 2-ethynylpiperidine-1-carboxylate* (**5a**) Yield 30%, a colorless oil; ^1^H-NMR (CDCl_3_) *δ* 5.06 (brs, 1H), 3.90 (d, *J* = 9.8 Hz, 1H), 2.99 (brs, 1H), 2.24 (s, 1H), 1.79–1.70 (m, 2H), 1.65–1.59 (m, 4H), 1.44 (s, 9H); ^13^C-NMR (CDCl_3_) *δ* 154.64, 82.16, 80.06, 84.13, 71.90, 30.44, 28.47, 25.31, 19.90; IR (ATR-IR) ν_max_ 3307, 2862, 2331, 1691, 1319, 1251, 1159, 921 cm^−1^; HRMS (ESI+) found: 154.0863 [calculated C_8_H_12_NO_2_ ([M + H − C_4_H_8_]^+^): 154.0869].

*tert-Butyl 2-ethynylazepane-1-carboxylate* (**5b**) Yield 28%, a yellow oil; ^1^H-NMR (500 MHz, CDCl_3_) *δ* 4.97 and 4.72 (m, 1H), 3.84 and 3.69 (d, *J* = 14.4 Hz, 1H), 3.00 (m, 1H), 2.22 (s, 1H), 2.17 (m, 1H), 1.66–1.24 (m, 7H), 1.46 (s, 9H); ^13^C-NMR (125 MHz, CDCl_3_) *δ* 155.34, 154.84, 84.22, 84.13, 79.92, 79.73, 70.34, 70.22, 47.36, 46.15, 42.54, 42.06, 35.54, 28.81, 28.75, 28.47, 28.37, 28.34, 24.81, 24.28; IR (ATR-IR) ν_max_ 3297, 2925, 1686, 1400, 1162 cm^−1^; HRMS (ESI+) found 168.1020 [calculated C_9_H_14_NO_2_ ([M + H − C_4_H_8_]^+^): 168.1019].

*tert-Butyl 2-ethynylazocane-1-carboxylate* (**5c**) Yield 84%, a colorless oil; ^1^H-NMR (500 MHz, CDCl_3_) *δ* 5.00 and 4.74 (m, 1H), 3.62 and 3.54 (m, 1H), 3.12 (m, 1H), 2.21 and 2.19 (d, *J* = 2.25 and 2.30 Hz, 1H), 1.89–1.85 (m, 3H), 1.67–1.25 (m, 7H), 1.47 (s, 9H); ^13^C-NMR (125 MHz, CDCl_3_) *δ* 155.45, 154.59, 83.57, 83.55, 79.88, 79.78, 70.33, 47.98, 46.83, 42.50, 42.20, 31.89, 31.32, 28.52, 28.50, 27.63, 26.85, 26.59, 26.49, 25.12, 24.72, 24.17, 23.52; IR (ATR-IR) ν_max_ 3299, 2924, 1686, 1400, 1157 cm^−1^; HRMS (ESI+) found: 182.1179 [calculated C_10_H_16_NO_2_ ([M + H − C_4_H_8_]^+^): 182.1176].

*tert-Butyl 2-((trimethylsilyl)ethynyl)piperidine-1-carboxylate* (**6a**) Yield 29%, a colorless oil; ^1^H-NMR (CDCl_3_) *δ* 5.04 (s, 1H), 3.90 (d, *J* = 12.1 Hz, 1H), 3.01 (m, 1H), 1.75–1.46 (m, 13H), 0.16 (s, 9H); ^13^C-NMR (CDCl_3_) *δ* 154.80, 104.29, 88.63, 80.00, 45.21, 40.46, 30.68, 28.56, 28.50, 25.48, 20.07, 0.19; IR (ATR-IR) ν_max_ 2954, 2163, 1678, 1404, 1249, 1147, 834 cm^−1^; HRMS (ESI+) found 226.1263 [calculated for C_11_H_20_NO_2_Si ([M + H − C_4_H_8_]^+^): 226.1258].

*tert-Butyl 2-((trimethylsilyl)ethynyl)azepane-1-carboxylate* (**6b**) Yield 38%, a colorless oil; ^1^H-NMR (CDCl_3_) *δ* 5.01 and 4.72 (m, 1H), 3.83 and 3.68 (d, *J* = 14.7 and 14.8 Hz, 1H), 3.04 (m, 1H), 2.09 (m, 1H), 1.67–1.32 (m, 7H), 1.47 (s, 9H), 0.15 (s, 9H); ^13^C-NMR (CDCl_3_) *δ* 155.38, 155.09, 106.15, 87.04, 86.70, 79.87, 79.66, 48.44, 46.97, 42.58, 42.18, 36.00, 35.50, 28.65, 28.58, 28.12, 25.05, 24.24, 0.12; IR (ATR-IR) ν_max_ 2931, 2174, 1689, 1161, 841 cm^−1^; HRMS (ESI+) found 240.1427 [calculated C_12_H_22_NO_2_Si ([M + H − C_4_H_8_]^+^): 240.1414].

*tert-Butyl 2-((trimethylsilyl)ethynyl)azocane-1-carboxylate* (**6c**) Yield 36%, a colorless oil; ^1^H-NMR (CDCl_3_) *δ* 5.01 and 4.70 (m, 1H), 3.55 (m, 1H), 3.14 (m, 1H), 1.89–1.81 (m, 3H), 1.73–1.44 (m, 10H), 1.47 (s, 9H), 0.14 and 0.13 (s, 9H); ^13^C-NMR (CDCl_3_) *δ* 155.50, 154.82, 87.05, 86.88, 79.84, 79.71, 48.92, 47.72, 42.83, 42.29, 32.33, 32.03, 31.71, 28.63, 28.61, 27.69, 27.51, 26.98, 26.86, 26.75, 25.00, 24.39, 23.53, 0.13, −0.17; IR (ATR-IR) ν_max_ 2928, 2169, 1687, 846 cm^−1^; HRMS (ESI+) found 254.1573 [calculated C_13_H_24_NO_2_Si ([M + H − C_4_H_8_]^+^): 254.1571].

*tert-Butyl 2-((trimethylsilyl)ethynyl)azonane-1-carboxylate* (**6d**) Yield 64%, a colorless oil; ^1^H-NMR (CDCl_3_) *δ* 5.04 and 4.72 (m, 1H), 3.51 (m, 1H), 3.15 and 3.08 (m, 1H), 1.82–1.44 (m, 21H), 0.12 (s, 9H); ^13^C-NMR (CDCl_3_) *δ* 156.20, 155.33, 105.40, 87.46, 79.85, 49.98, 48.89, 43.94, 32.83, 31.96, 28.64, 27.28, 26.64, 26.58, 25.94, 24.95, 24.89, 24.78, 0.12; IR (ATR-IR) ν_max_ 2921, 1689, 1162, 847 cm^−1^; HRMS (ESI+) found 268.1731 [calculated C_14_H_26_NO_2_Si ([M + H − C_4_H_8_]^+^): 268.1727].

*tert-Butyl 2-(prop-1-yn-1-yl)piperidine-1-carboxylate* (**7a**) Yield 35%, a colorless oil; ^1^H-NMR (CDCl_3_) *δ* 5.01 (s, 1H), 3.89 (d, *J* = 11.2 Hz, 1H), 3.03 (t, *J* = 12.3 Hz, 1H), 1.81 (d, *J* = 2.3, 3H), 1.67–1.57 (m, 6H), 1.44 (s, 9H); ^13^C-NMR (CDCl_3_) *δ* 154.74, 79.78, 79.61, 44.41, 40.39, 31.02, 28.57, 25.55, 20.12, 3.64; IR (ATR-IR) ν_max_ 2932, 1689, 1401, 1258, 1155, 1028, 861 cm^−1^; HRMS (ESI+) found: 168.1018 [calculated for C_9_H_14_NO_2_ ([M + H − C_4_H_8_]^+^) 168.1019].

*tert-Butyl 2-(prop-1-yn-1-yl)azepane-1-carboxylate* (**7b**) Yield 89%, a yellow oil; ^1^H-NMR (CDCl_3_) *δ* 4.90 and 4.68 (m, 1H), 3.81 and 3.66 (d, *J* = 14.6 Hz, 1H), 2.99 (m, 1H), 2.09 (m, 1H), 1.78 (m, 3H), 1.62–1.27 (m, 7H), 1.45 (s, 9H); ^13^C-NMR (CDCl_3_) *δ* 155.49, 155.04, 79.66, 79.50, 79.44, 79.35, 78.06, 47.72, 46.53, 42.44, 42.01, 36.18, 36.13, 28.92, 28.86, 28.60, 28.40, 28.25, 24.83, 24.37, 3.72, 3.58; IR (ATR-IR) ν_max_ 2925, 1686, 1401, 1161, 976 cm^−1^; HRMS (ESI+) found 182.1174 [calculated C_10_H_16_NO_2_ ([M + H − C_4_H_8_]^+^): 182.1176].

*tert-Butyl 2-(prop-1-yn-1-yl)azocane-1-carboxylate* (**7c**) Yield 82%, a colorless oil; ^1^H-NMR (CDCl_3_) *δ* 4.92 and 4.68 (m, 1H), 3.58 and 3.50 (m, 1H), 3.08 (m, 1H), 1.76–1.44 (m, 22H); ^13^C-NMR (CDCl_3_) *δ* 155.58, 154.77, 79.59, 79.52, 78.92, 78.83, 78.17, 78.03, 48.35, 47.22, 42.44, 42.13, 32.43, 31.75, 28.63, 27.78, 26.94, 26.85, 26.68, 25.03, 24.79, 24.21, 23.62, 3.63; IR (ATR-IR) ν_max_ 2924, 1685, 1467, 1400, 1156, 934 cm^−1^; HRMS (ESI+) found 196.1341 [calculated C_11_H_18_NO_2_ ([M + H − C_4_H_8_]^+^): 196.1332].

*tert-Butyl 2-(prop-1-yn-1-yl)azonane-1-carboxylate* (**7d**) Yield 80%, a colorless oil; ^1^H-NMR (CDCl_3_) *δ* 4.97 and 4.73 (m, 1H), 3.55–3.44 (m, 1H), 3.06 (m, 1H), 1.77–1.45 (m, 24H); ^13^C-NMR (CDCl_3_) *δ* 156.29, 155.30, 79.73, 79.67, 78.76, 78.70, 78.62, 78.50, 49.42, 48.36, 44.68, 43.84, 39.48, 32.98, 32.05, 29.81, 29.38, 28.65, 27.51, 27.34, 26.77, 26.62, 26.04, 25.39, 25.08, 24.83, 24.76, 23.46, 14.29, 13.82, 8.86, 3.62, 3.55; IR (ATR-IR) ν_max_ 2918, 1687, 1399, 1157, 768 cm^−1^; HRMS (ESI+) found 210.1493 [calculated C_12_H_20_NO_2_ ([M + H − C_4_H_8_]^+^) 210.1489].

*tert-Butyl 2-(phenylethynyl)piperidine-1-carboxylate* (**8a**) Yield 31%, a colorless oil; ^1^H-NMR (CDCl_3_) *δ* 7.41 (m, 2H), 7.30 (m, 3H), 5.28 (s, 1H), 3.97 (d, *J* = 12 Hz, 1H), 3.11 (t, *J* = 11.7 Hz, 1H), 1.84 (m, 2H), 1.68–1.48 (m, 13H); ^13^C-NMR (CDCl_3_) *δ* 154.78, 131.85, 128.36, 128.20, 123.27, 87.81, 84.22, 80.05, 44.92, 40.65, 30.89, 28.58, 25.53, 20.29; IR (ATR-IR) ν_max_ 2932, 1681, 1401, 1149, 753 cm^−1^; HRMS (ESI+) found 230.1189 [calculated C_14_H_16_NO_2_ ([M + H − C_4_H_8_]^+^): 230.1176].

*tert-Butyl 2-(phenylethynyl)azepane-1-carboxylate* (**8b**) Yield 88%, a colorless oil; ^1^H-NMR (CDCl_3_) *δ* 7.40 (m, 2H), 7.28 (m, 3H), 5.23 and 4.96 (m, 1H), 3.90 and 3.87 (d, *J* = 14.4 Hz, 1H), 3.13 (m, 1H), 2.24 (m, 1H), 1.78–1.40 (m, 16H); ^13^C-NMR (CDCl_3_) *δ* 155.45, 155.09, 131.86, 131.72, 128.34, 128.26, 128.14. 128.09, 123.32, 123.27, 89.66, 89.59, 82.66, 82.53, 79.92, 79.71, 48.28, 46.88, 42.74, 42.32, 36.02, 35.72, 28.81, 28.61, 28.54, 28.31, 25.07, 24.40; IR (ATR-IR) ν_max_ 2931, 1676, 1393, 1149, 764 cm^−1^; HRMS (ESI+) found 244.1335 [calculated C_15_H_18_NO_2_ ([M + H − C_4_H_8_]^+^): 244.1332].

*tert-Butyl 2-(phenylethynyl)azocane-1-carboxylate* (**8c**) Yield 68%, a colorless oil; ^1^H-NMR (CDCl_3_) *δ* 7.39 (m, 2H), 7.28 (m, 3H), 5.24–4.94 (m, 1H), 3.58 (m, 1H), 3.21 (m, 1H), 1.69–1.49 (m, 19H); ^13^C-NMR (CDCl_3_) *δ* 155.55, 154.79, 131.82, 131.69, 128.31, 128.24, 128.13, 128.08, 123.25, 123.17, 89.10, 82.68, 79.84, 79.72, 48.81, 47.60, 42.84, 42.39, 32.30, 31.67, 28.62, 27.75, 26.99, 26.82, 26.71, 25.12, 24.67, 24.36, 23.57; IR (ATR-IR) ν_max_ 2925, 1685, 1400, 1156, 760 cm^−1^; HRMS (ESI+) found 258.1495 [calculated C_16_H_20_NO_2_ ([M + H − C_4_H_8_]^+^): 258.1489].

*tert-Butyl 2-(phenylethynyl)azonane-1-carboxylate* (**8d**) Yield 81%, a colorless oil; ^1^H-NMR (CDCl_3_) *δ* 7.40 (m, 2H), 7.29 (m, 3H), 5.29 and 4.99 (m, 1H), 3.59 (m, 1H), 3.17 (m, 1H), 1.93–1.50 (m, 21H); ^13^C-NMR (CDCl_3_) *δ* 156.35, 155.38, 131.85, 131.74, 128.40, 128.33, 128.22, 128.16, 123.31, 123.20, 88.98, 88.84, 83.18, 80.07, 79.98, 49.93, 48.81, 44.12, 32.87, 31.94, 28.70, 27.39, 26.76, 26.63, 26.09, 25.25, 25.11, 24.91, 24.83; IR (thin film, neat) ν_max_ 2927, 1692, 1161, 772 cm^−1^; HRMS (ESI+) found 272.1648 [calculated C_17_H_22_NO_2_ ([M + H − C_4_H_8_]^+^): 272.1645].

## 4. Conclusions

In summary, we developed an efficient route for the synthesis of α-vinyl azacycles from 7- to 9-memebered lactams and α-acetylenyl azacycles from 6- to 9-membered lactams via *N*,*O*-acetal TMS ethers using organocopper and organostannane reagents, respectively. This procedure affords reasonable yields and may facilitate the concise and efficient synthesis of α-vinyl and α-acetylenyl azacycles to provide bioactive alkaloids and pharmaceuticals. 

## Figures and Tables

**Table 1:**
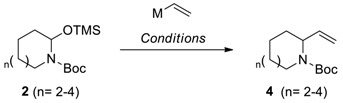
Vinylation of *N*,*O*-acetal TMS ethers.

Entry ^a^	n	M	Additive	Lewis Acid	Solvent	Yield ^b^
1	4	Li		BF_3_·OEt_2_	THF	ND ^c^
2	4	SiMe_3_		BF_3_·OEt_2_	CH_2_Cl_2_	ND ^c^
3	4	MgBr		BF_3_·OEt_2_	THF	ND ^c^
4	4	MgBr	CuBr·SMe_2_	BF_3_·OEt_2_	THF	63
5	4	MgBr	CuBr·SMe_2_	BF_3_·OEt_2_	Et_2_O	32
6	4	MgBr	CuI	BF_3_·OEt_2_	THF	ND ^c^
7	4	MgBr	CuOTf	BF_3_·OEt_2_	THF	ND ^c^
8	3	MgBr	CuBr·SMe_2_	BF_3_·OEt_2_	THF	60
9	2	MgBr	CuBr·SMe_2_	BF_3_·OEt_2_	THF	65
10	1	MgBr	CuBr·SMe_2_	BF_3_·OEt_2_	THF	ND ^c^

^a^ All reactions were performed using 0.3 mmol of **2** in dry solvent. See, Experimental section for a representative procedure. ^b^ Isolated yields. ^c^ Not detected.

**Table 2:**
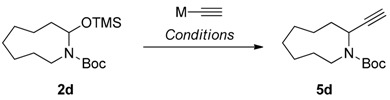
Optimization of the acetylenylation conditions for *N*,*O*-acetal TMS ethers.

Entry ^a^	M	Additive	Lewis acid	Solvent	Yield ^b^
1	MgBr	CuBr·SMe_2_	BF_3_·OEt_2_	THF	ND ^c^
2	MgBr		BF_3_·OEt_2_	THF	ND ^c^
3	Li		BF_3_·OEt_2_	THF	ND ^c^
4	SiMe_3_		BF_3_·OEt_2_	CH_2_Cl_2_	ND ^c^
5	*n*-Bu_3_Sn		BF_3_·OEt_2_	CH_2_Cl_2_	58
6	*n*-Bu_3_Sn		BF_3_·OEt_2_	THF	ND ^c^
7	*n*-Bu_3_Sn		BF_3_·OEt_2_	PhCH_3_	37
8	*n*-Bu_3_Sn		BF_3_·OEt_2_	Et_2_O	29
9	*n*-Bu_3_Sn		SnCl_4_	CH_2_Cl_2_	ND ^c^
10	*n*-Bu_3_Sn		TiCl_4_	CH_2_Cl_2_	ND ^c^
11	*n*-Bu_3_Sn		TMSOTf	CH_2_Cl_2_	46

^a^ All reactions were performed using 0.2 mmol of 2d in dry solvent (2 mL) by adding the additive (if any) at −50 °C. The nucleophile was then added at the same temperature followed by the addition of BF_3_·OEt_2_ at −78 °C and stirring for 1 h. The reaction mixture was slowly warmed to r.t. over 2 h before quenching with Et_3_N. ^b^ Isolated yields. ^c^ Not detected.

**Table 3:**
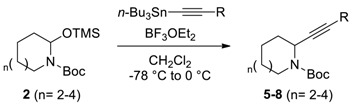
Acetylenylation of *N*,*O*-acetal TMS ethers.

Entry ^a^	n	R	Yield ^b^
1	1	H	30
2	1	SiMe_3_	29
3	1	Me	35
4	1	Ph	31
5	2	H	28
6	2	SiMe_3_	38
7	2	Me	89
8	2	Ph	88
9	3	H	84
10	3	SiMe_3_	36
11	3	Me	82
12	3	Ph	68
13	4	H	58
14	4	SiMe_3_	64
15	4	Me	80
16	4	Ph	81

^a^ All reactions were performed using 0.2 mmol of 2a–d in dry solvent (2 mL) by first adding the nucleophile at −78 °C followed by adding BF_3_·OEt_2_ and stirring for 1 h. The mixture was then slowly warmed to r.t. over 1 h before quenching with Et_3_N. ^b^ Isolated yields.

## Supplementary Materials

The following are available online at http://www.mdpi.com/1420-3049/23/11/3023/s1. The ^1^H and ^13^C NMR spectra of **4b**–**8d** can be found in the SI.
