# Supplementary material for: Conversion of Medium-Sized Lactams to α-Vinyl or α-Acetylenyl Azacycles via N,O-Acetal TMS Ethers
Source: Molecules. 2018 Nov 19;23(11):3023. doi: 10.3390/molecules23113023 (PMC6278647; doi:10.3390/molecules23113023)

Supplementary Information

Conversion of Medium-sized Lactam to α-Vinylated or α-Acetylenylated Azacycles *via N,O*-Acetal TMS Ethers

Minjun Kim,[1,§] Jaebong Jang,[2,3,§] Goyoung Choi,[1] Sungkyun Chung,[4] Changjin Lim,[4] Joonseong Hur,[5] Hyun Su Kim,[4] Younghwa Na[4], Woo Sung Son[4], Young-Ger Suh,[d] Jong-Wha Jung,[1,*] and Seok-Ho Kim,[4,*]

[a] College of Pharmacy, Research Institute of Pharmaceutical Sciences, Kyungpook National University, Daegu 41566, Republic of Korea

[b] Department of Cancer Biology, Dana-Farber Cancer Institute, Boston, MA 02215, USA

[c] Department of Biological Chemistry and Molecular Pharmacology, Harvard Medical School, Boston, MA 02215, USA

[d] Department of Pharmacy, College of Pharmacy and Institute of Pharmaceutical Sciences, CHA University, 120 Haeryong-ro, Pocheon 11160, Republic of Korea

[e] College of Pharmacy, Seoul National University, 1 Gwanak-ro, Gwanak-gu, Seoul 08826, Republic of Korea

[§]These authors contributed equally to this work.

Correspondence to: J.-W. Jung (jungj@knu.ac.kr) and S.-H. Kim (ksh3410@cha.ac.kr)

Table of Contents

Copies of ^{1}H NMR and ^{13}C NMR Spectra-- S1

^{1}H- and ^{13}C-NMR Spectra

tert-Butyl 2-vinylazepane-1-carboxylate (**4a**)

^{1}H-NMR

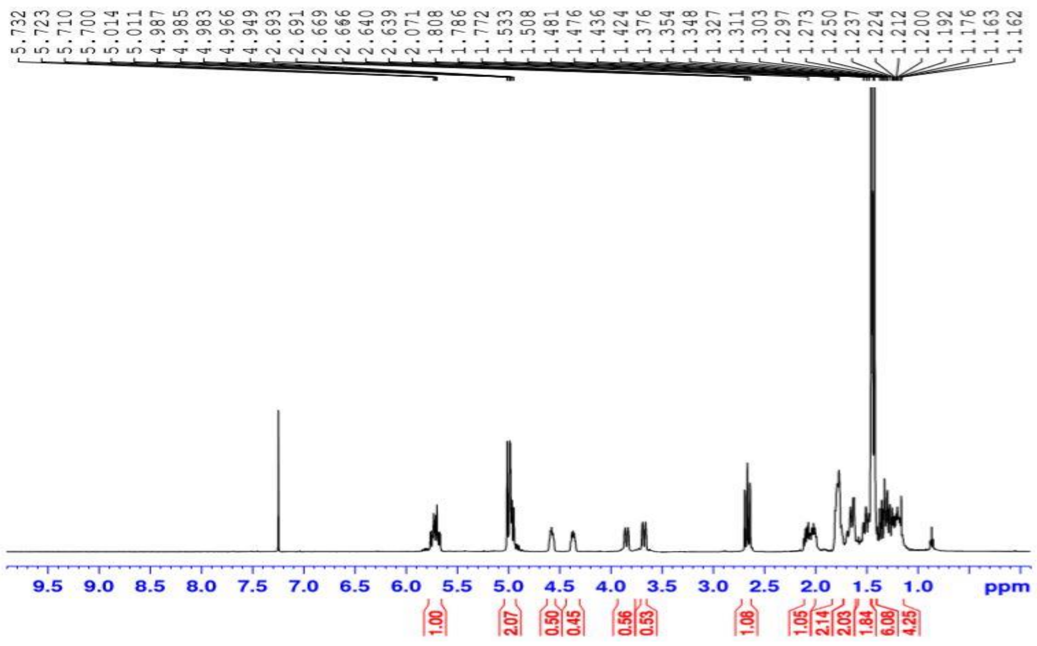

^{13}C-NMR

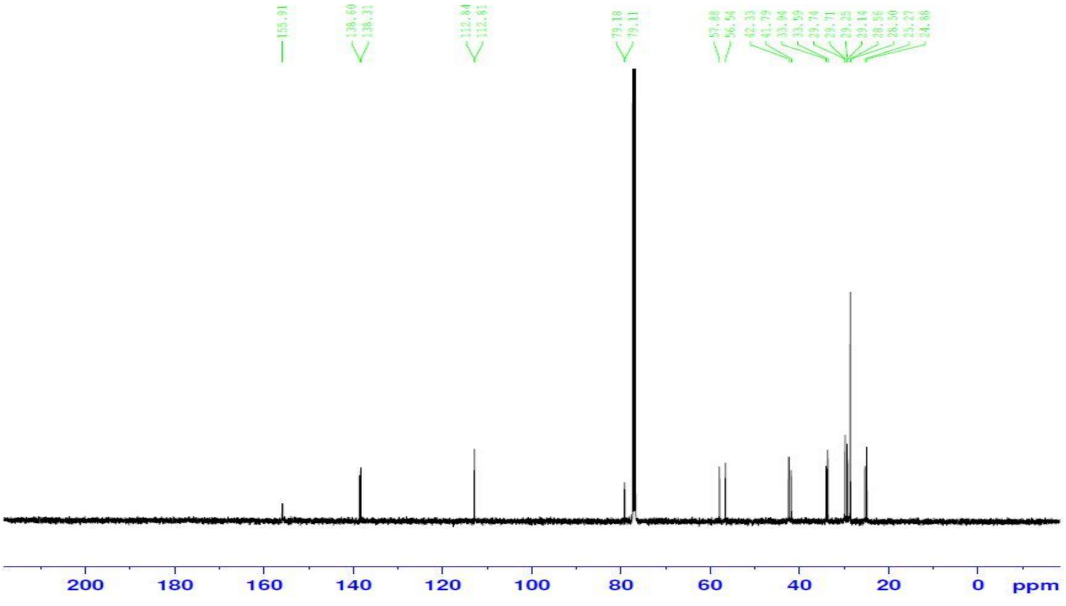

tert-Butyl 2-vinylazocane-1-carboxylate (**4b**)

^{1}H-NMR

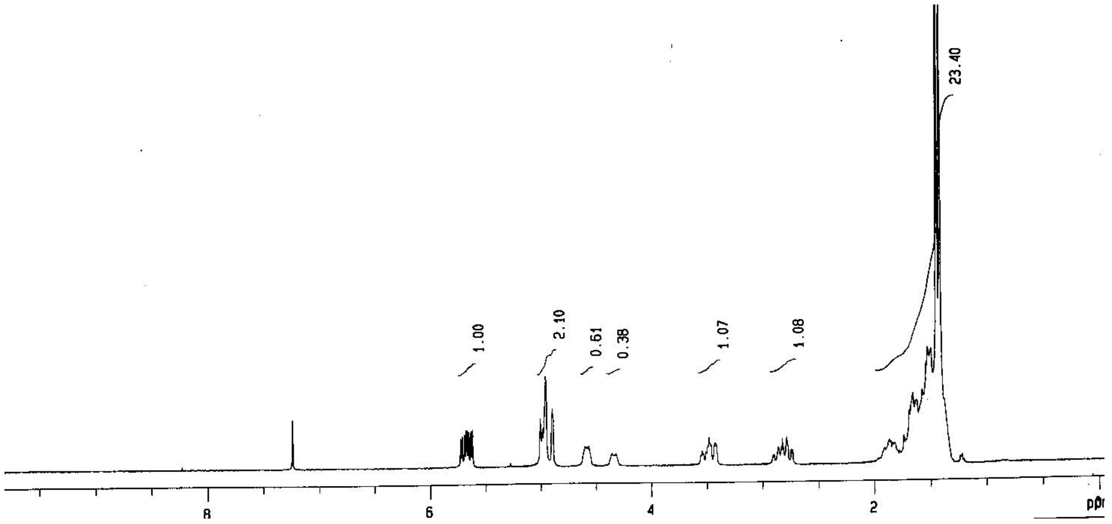

^{13}C-NMR

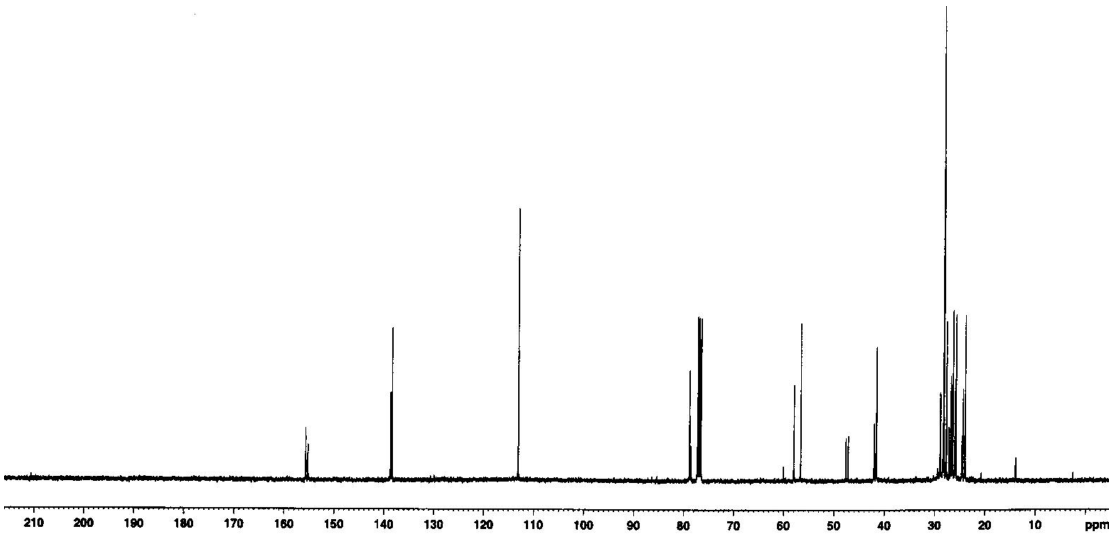

tert-Butyl 2-vinylazonane-1-carboxylate (**4c**)

^{1}H-NMR

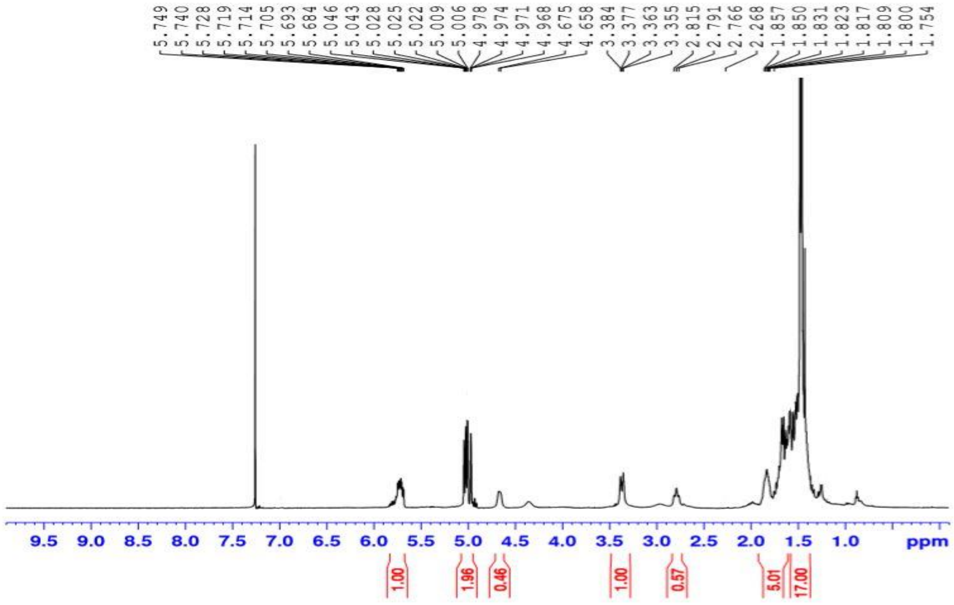

^{13}C-NMR

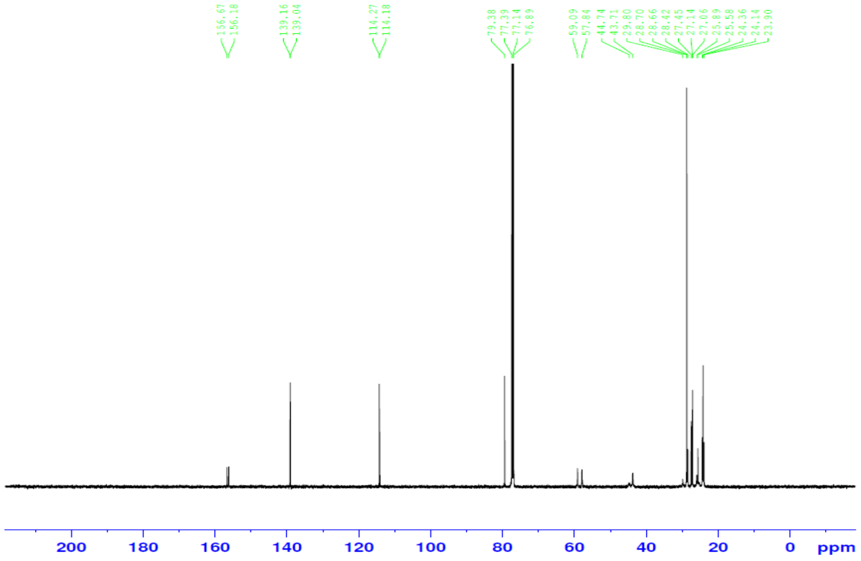

tert-Butyl 2-ethynylpiperidine-1-carboxylate(**5a**)

^{1}H-NMR
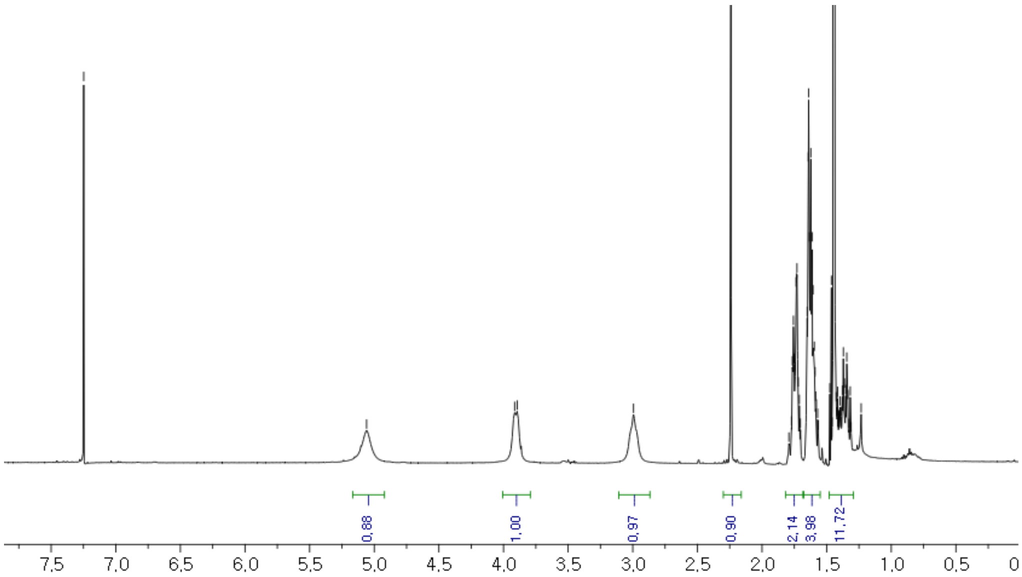
^{13}C-NMR
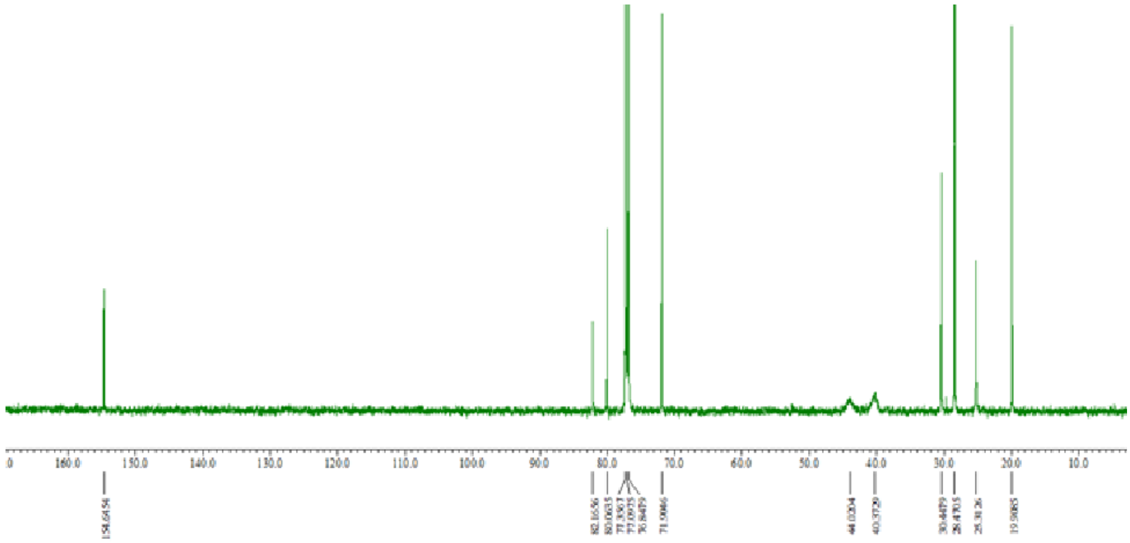

tert-Butyl 2-ethynylazepane-1-carboxylate(**5b**)

^{1}**H-NMR**

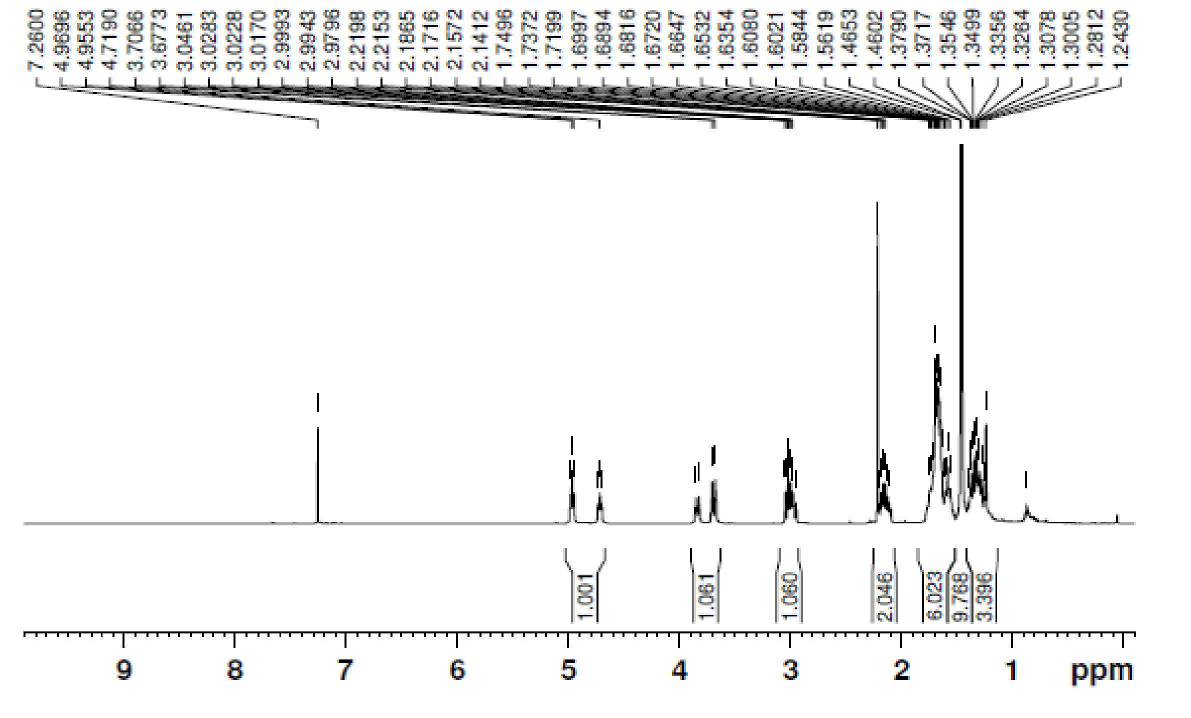

^{13}**C-NMR**

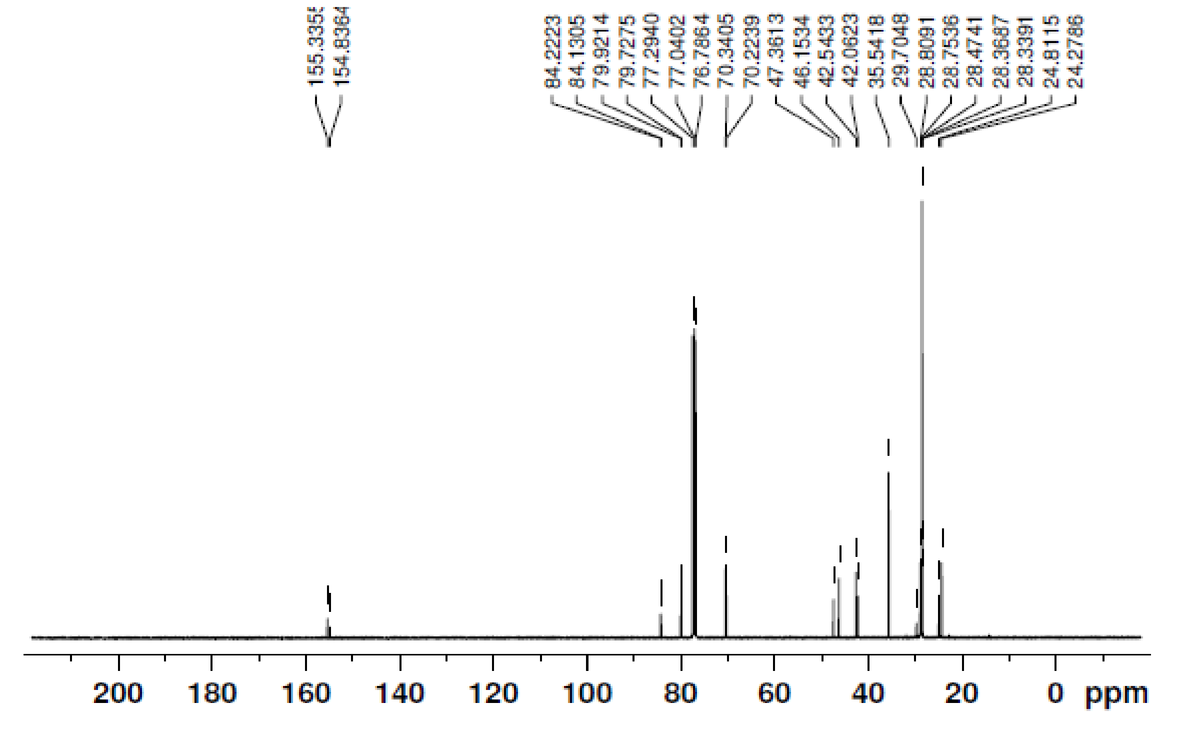

tert-Butyl 2-ethynylazocane-1-carboxylate (**5c**)

^{1}H-NMR

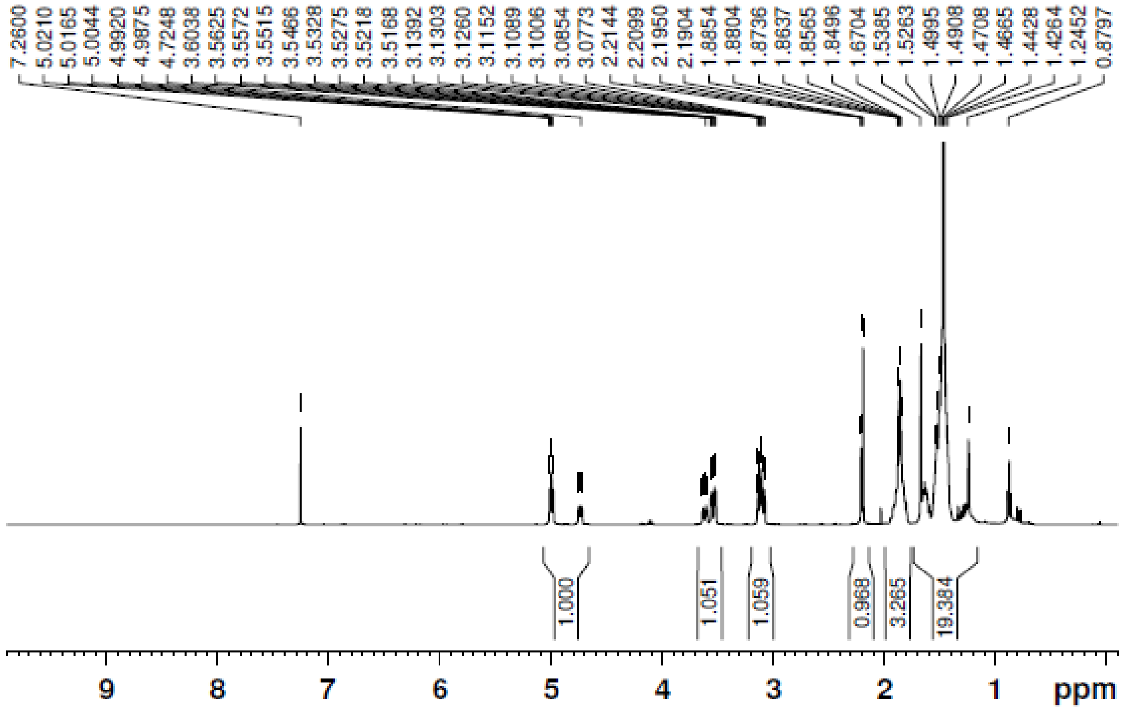

^{13}C-NMR

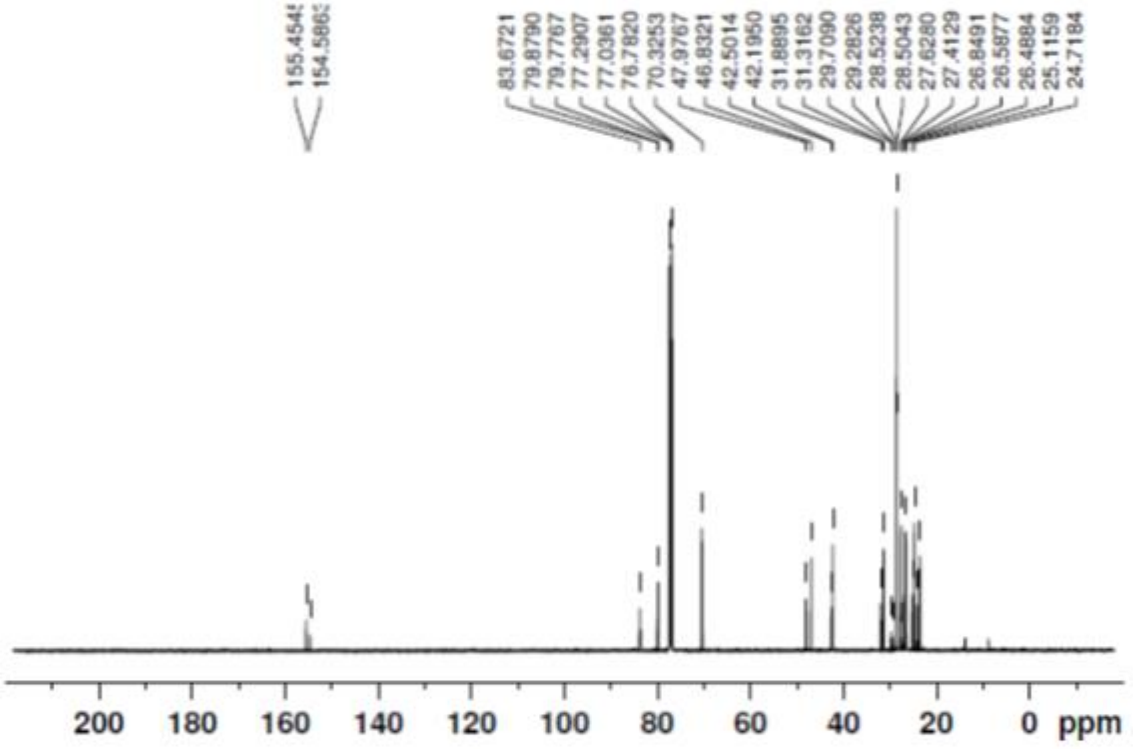

tert-Butyl 2-ethynylazonane-1-carboxylate (**5d**)

^{1}**H-NMR**

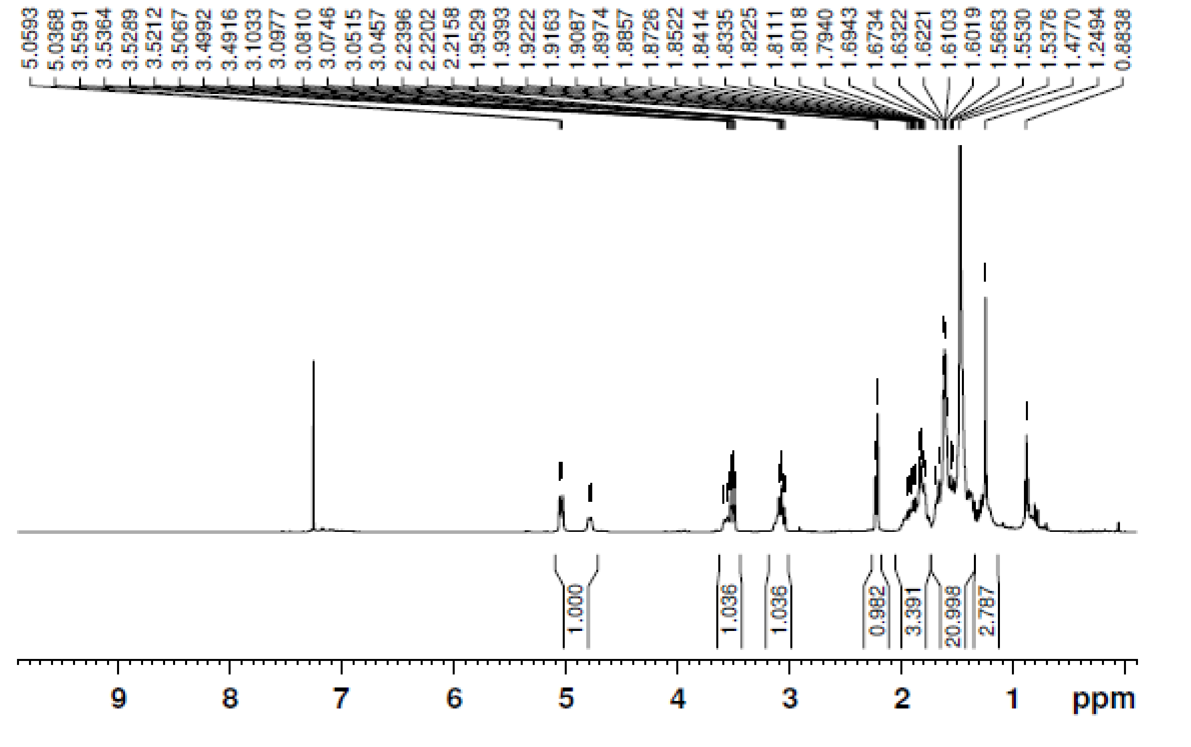

^{13}**C-NMR**

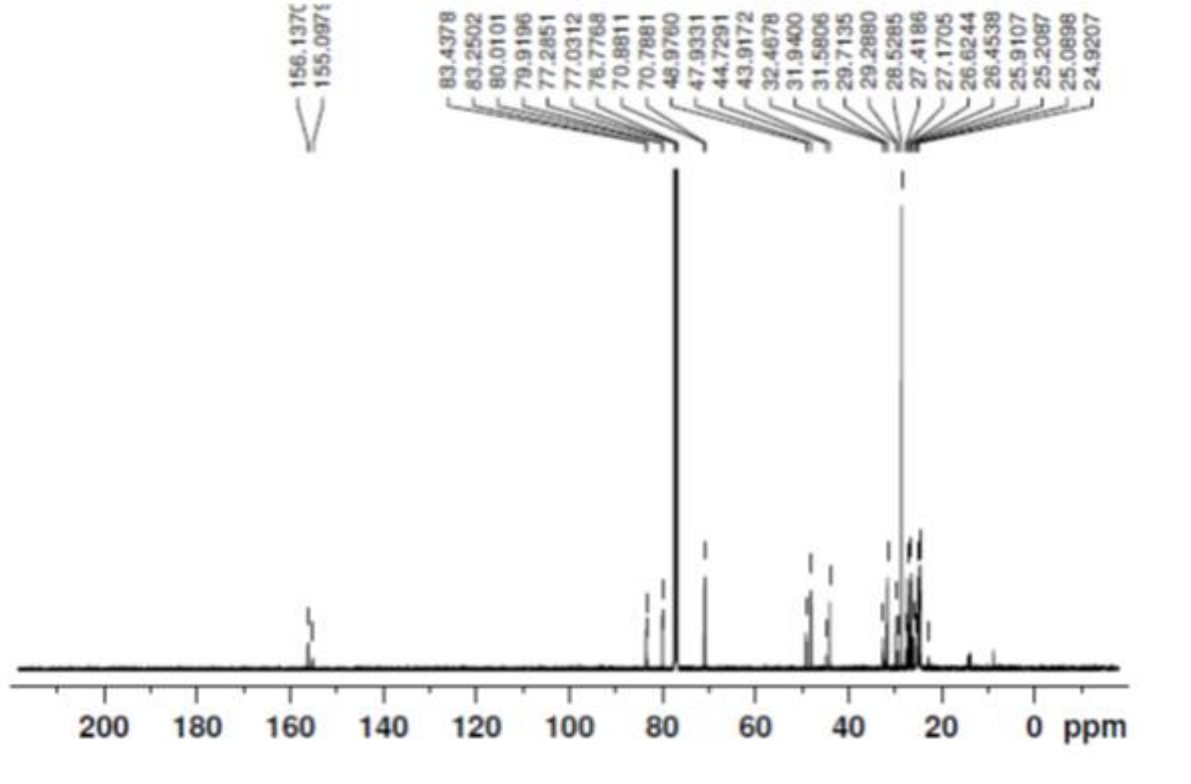

tert-Butyl 2- ((trimethylsilyl)ethynyl)piperidine-1-carboxylate (**5e**)

^{1}H-NMR

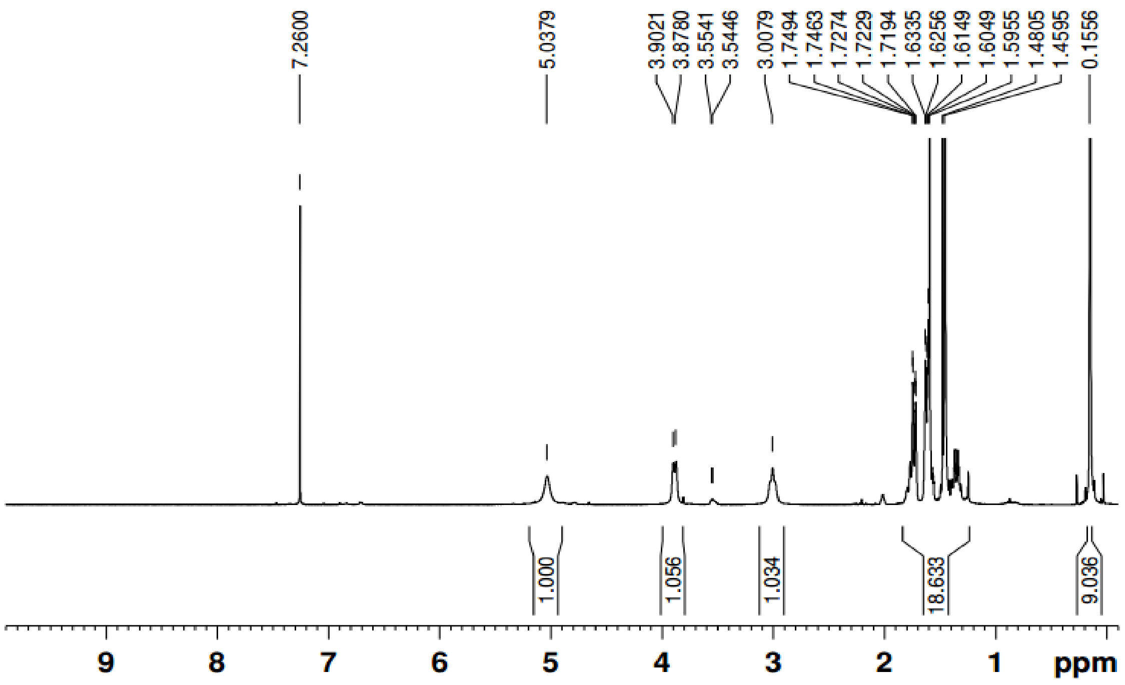

^{13}C-NMR

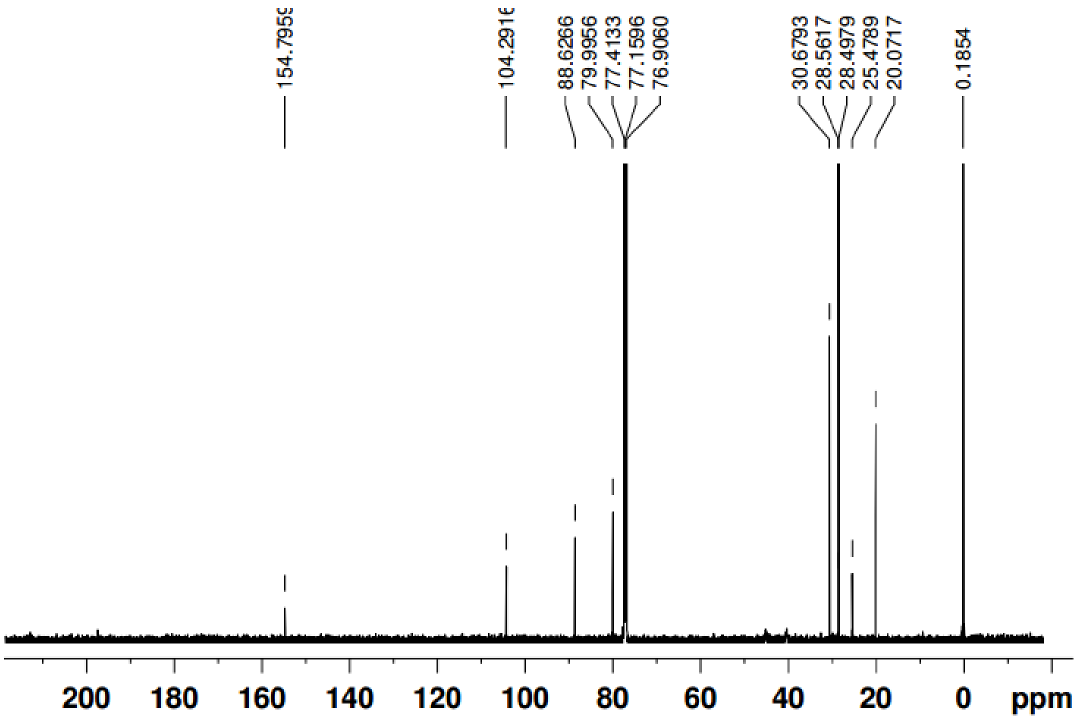

tert-Butyl 2-((trimethylsilyl)ethynyl)azepane-1-carboxylate (**5f**)

^{1}H-NMR

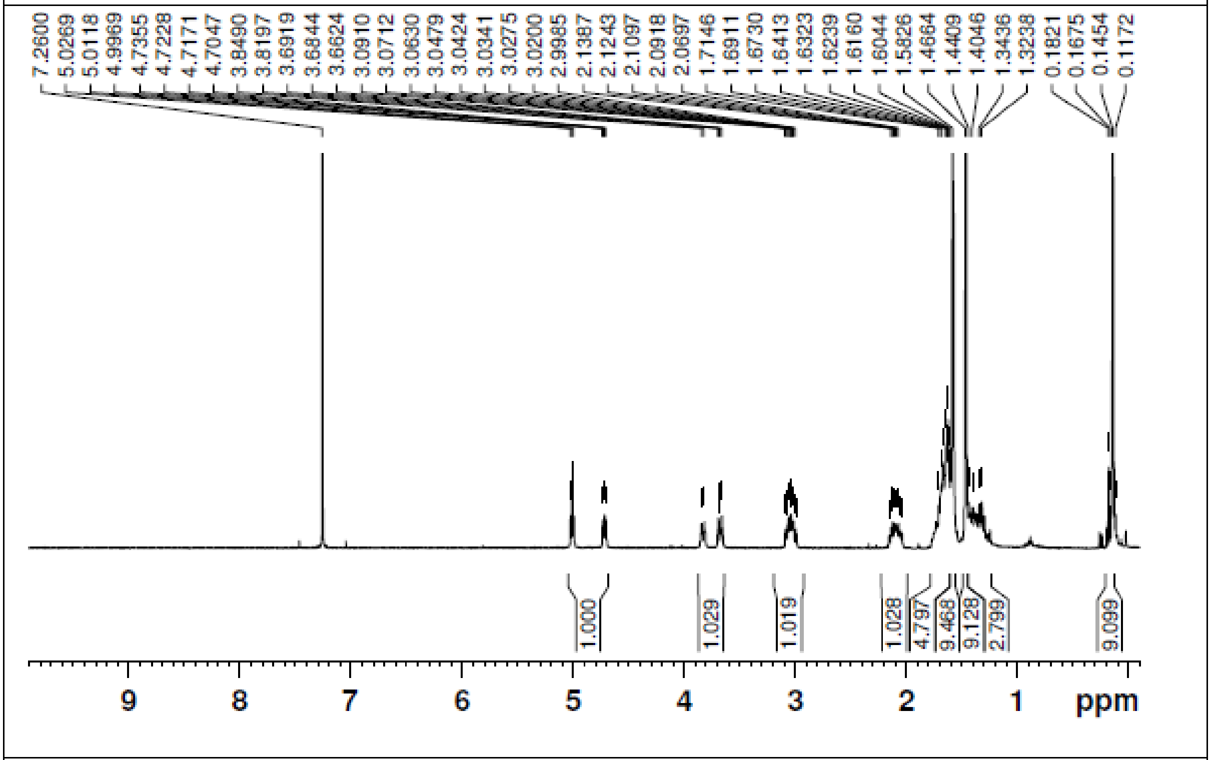

^{13}C-NMR

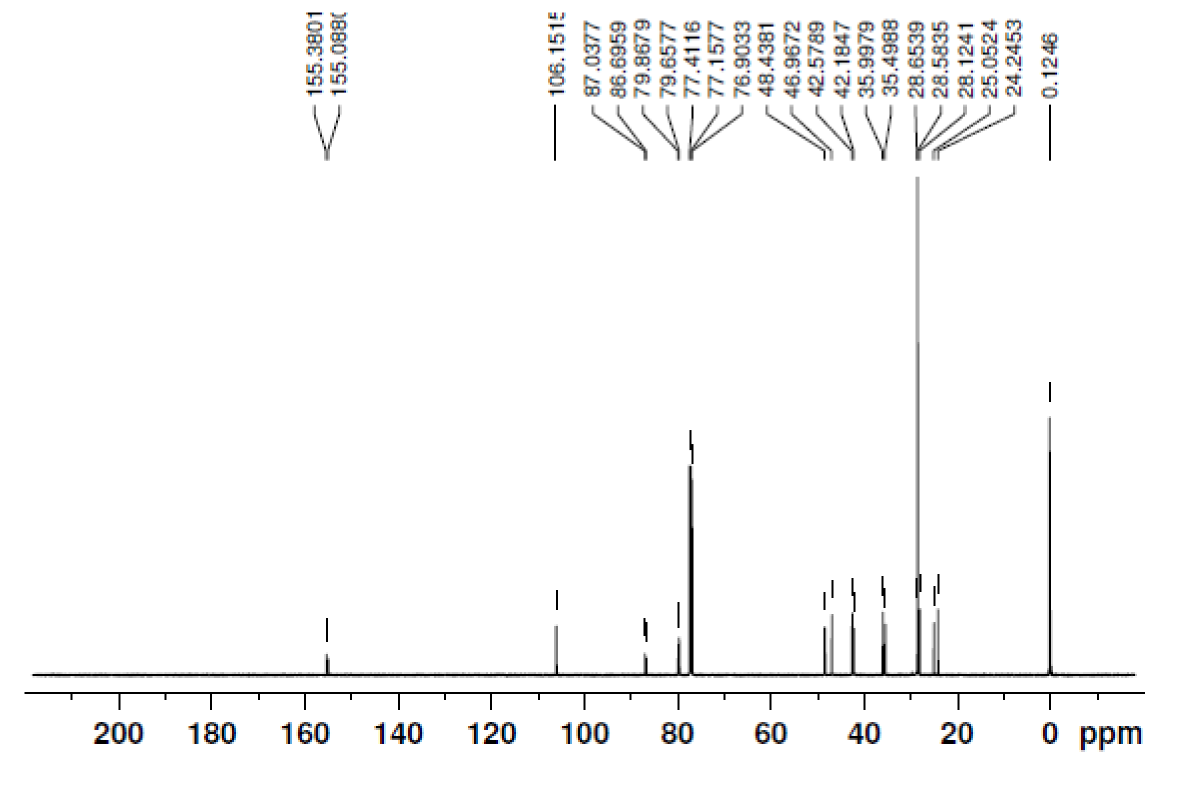

tert-Butyl 2-((trimethylsilyl)ethynyl)azocane-1-carboxylate(**5g**)

^{1}H-NMR

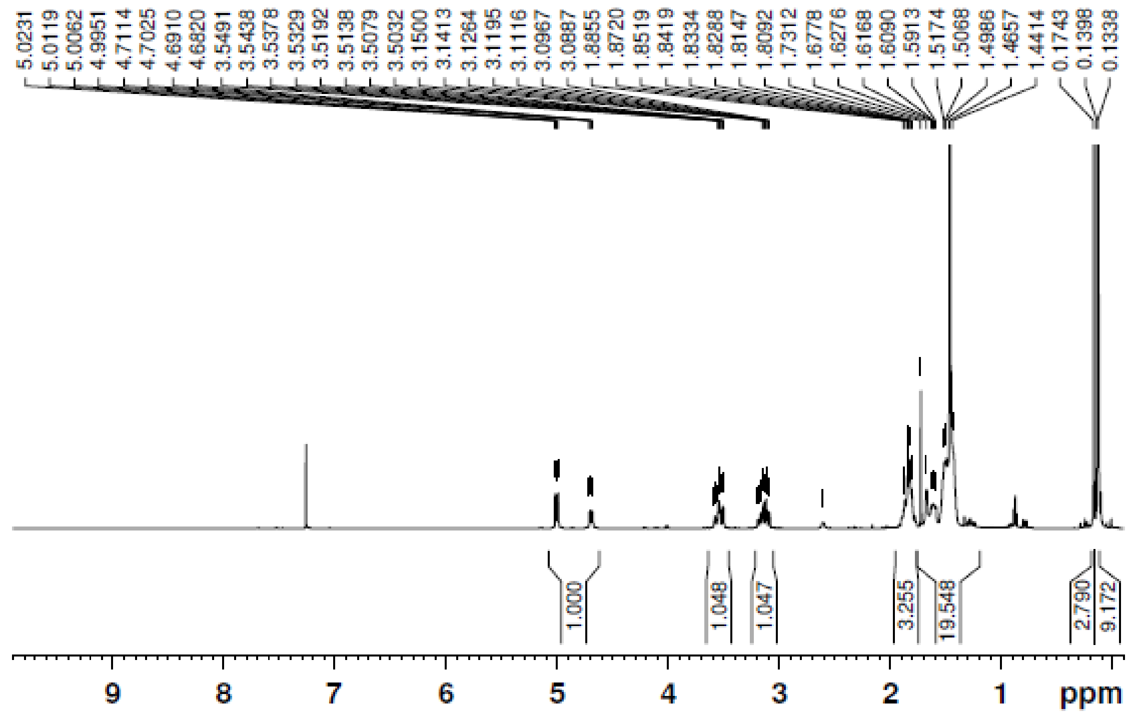

^{13}C-NMR

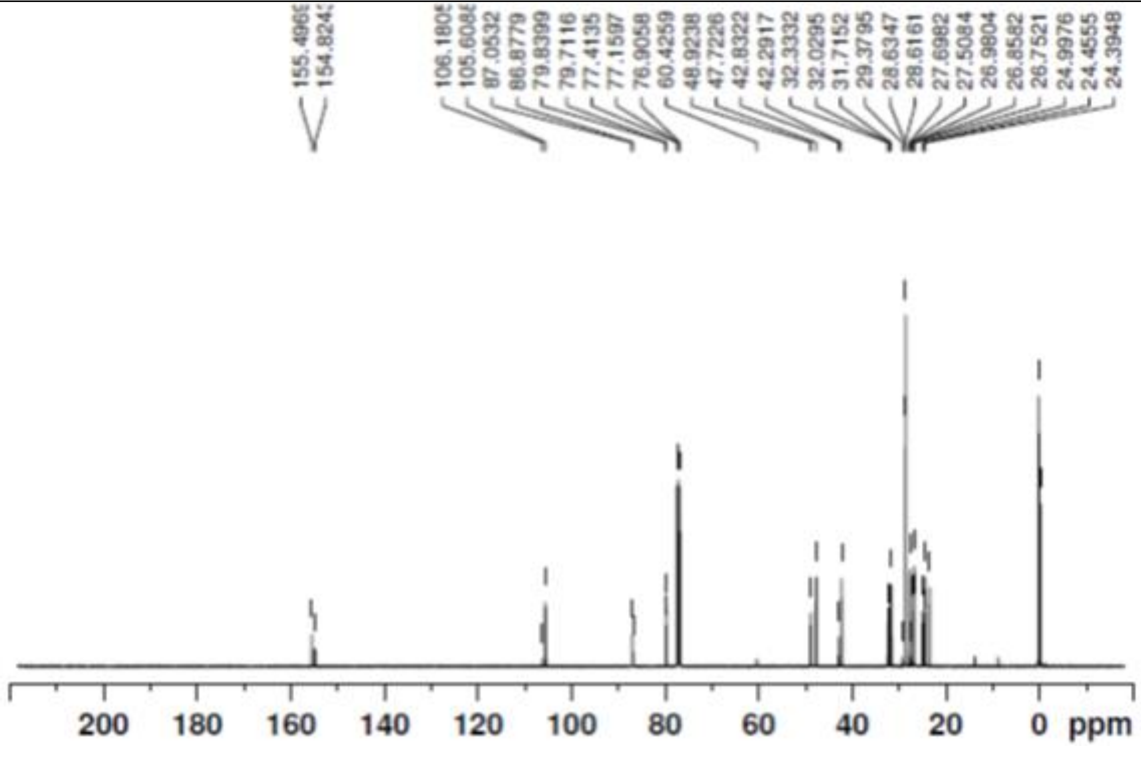

tert-Butyl 2-((trimethylsilyl)ethynyl)azonane-1-carboxylate (**5h**)

^{1}H-NMR

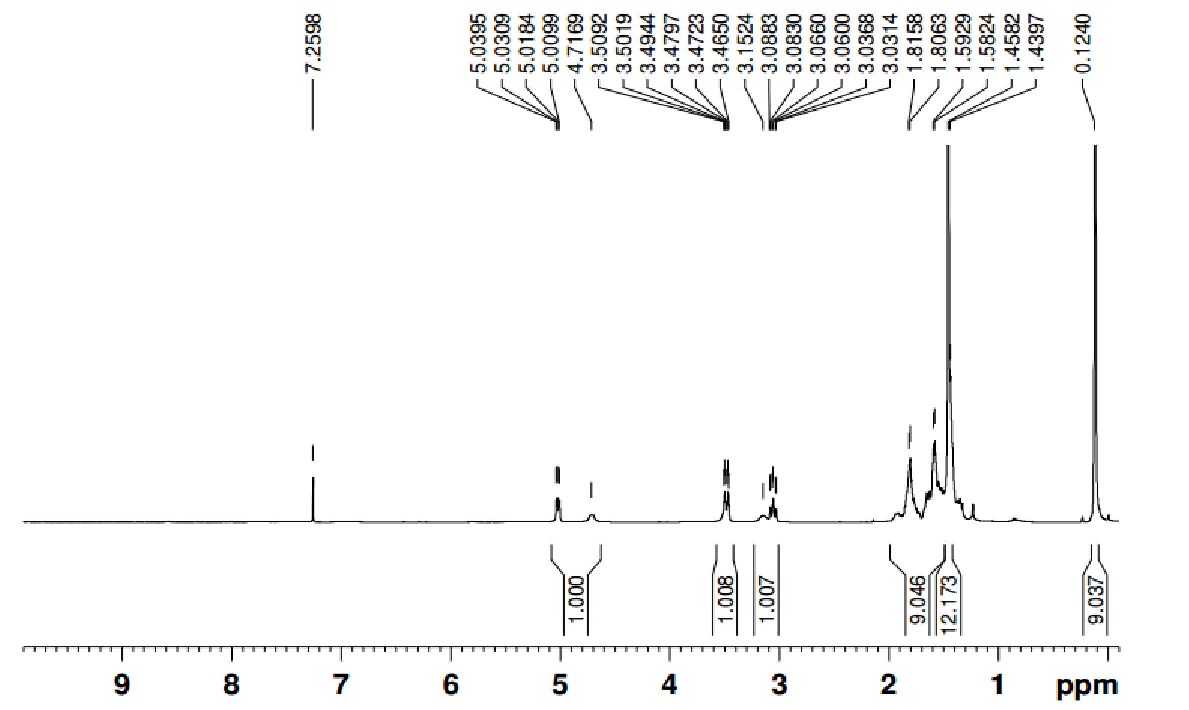

^{13}C-NMR

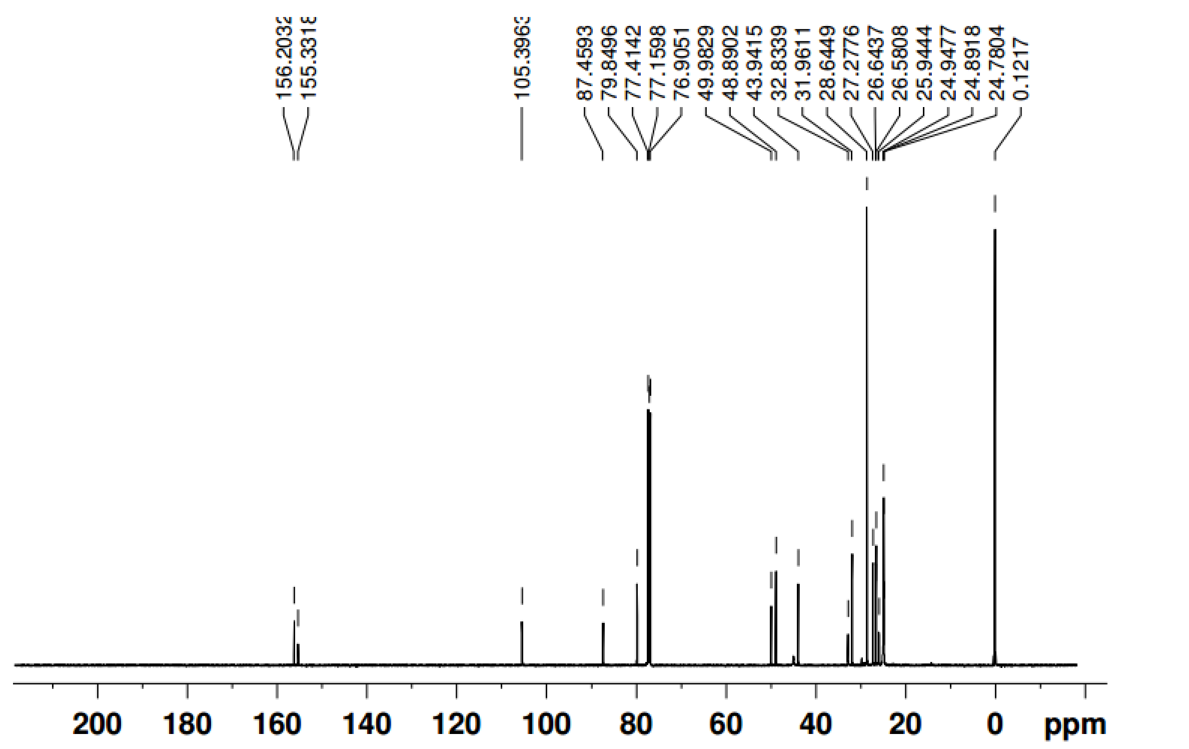

tert-Butyl 2-(prop-1-yn-1-yl)piperidine-1-carboxylate (**5i**)

^{1}H-NMR

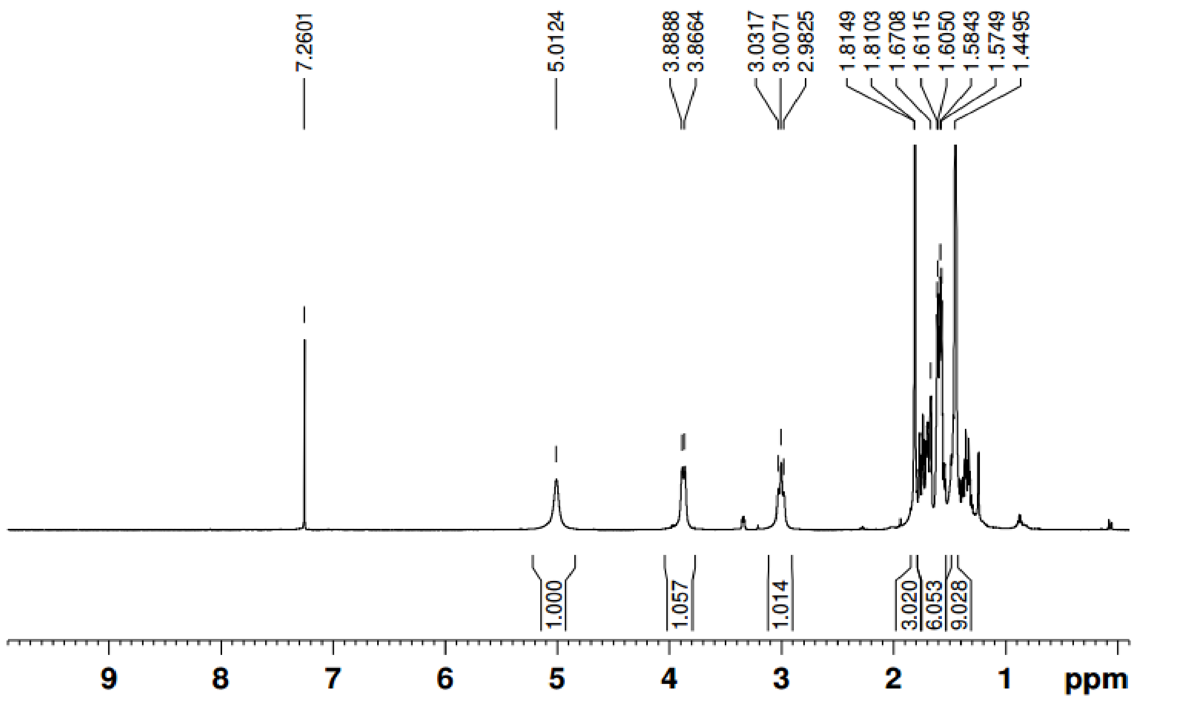

^{13}C-NMR

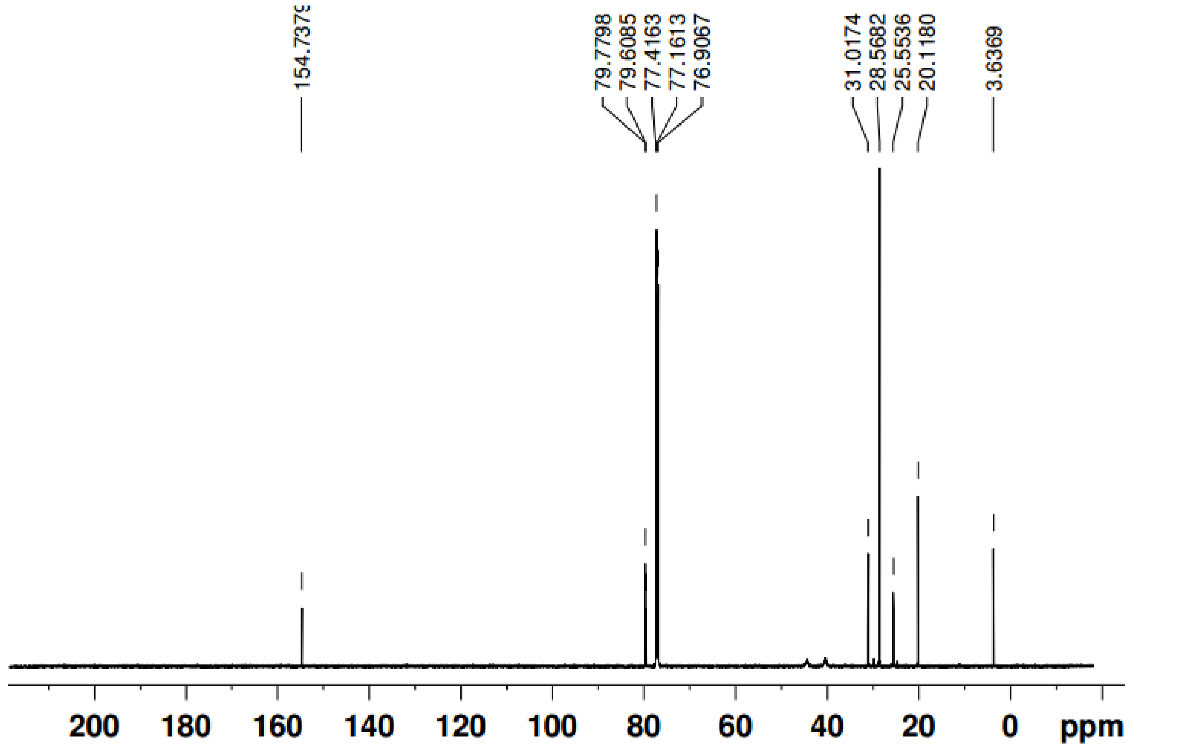

tert-Butyl 2-(prop-1-yn-1-yl)azepane-1-carboxylate (**5j**)

^{1}H-NMR

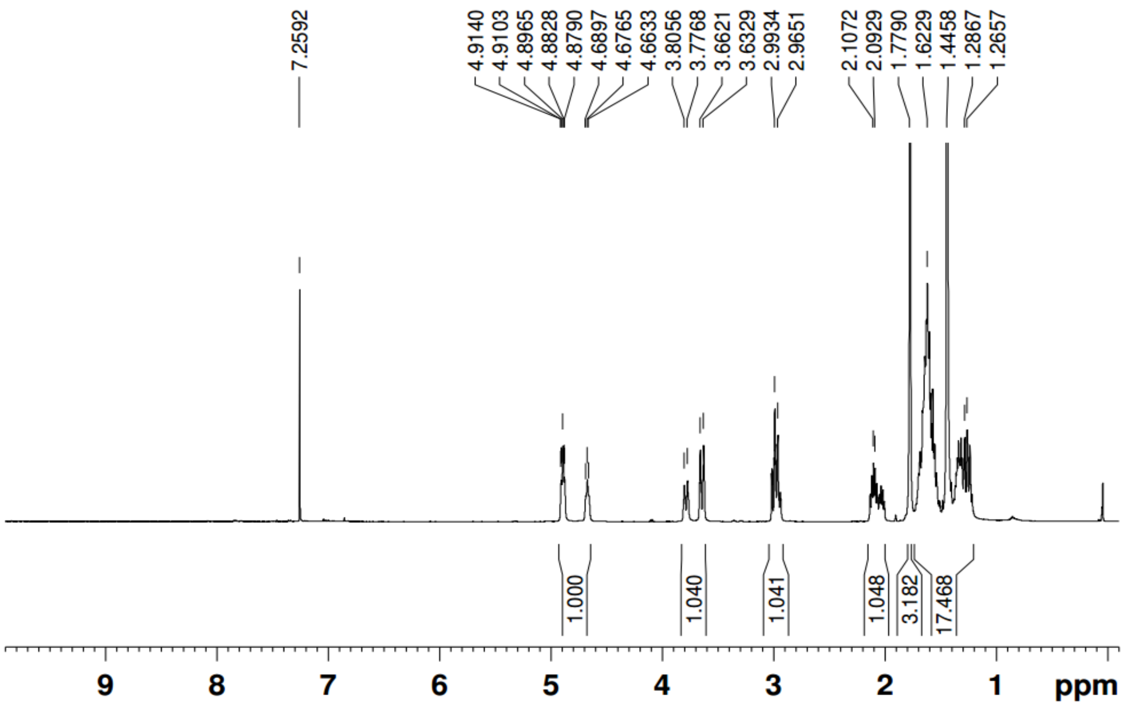

^{13}C-NMR

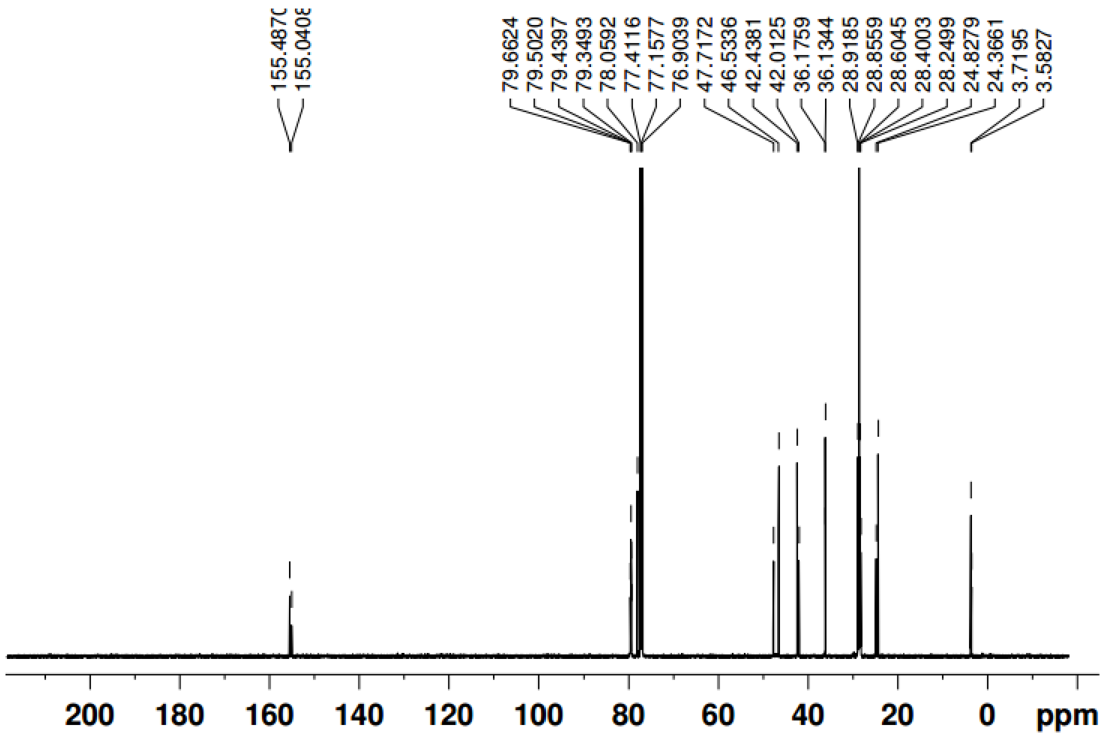

tert-Butyl 2-(prop-1-yn-1-yl)azocane-1-carboxylate (**5k**)

^{1}H-NMR

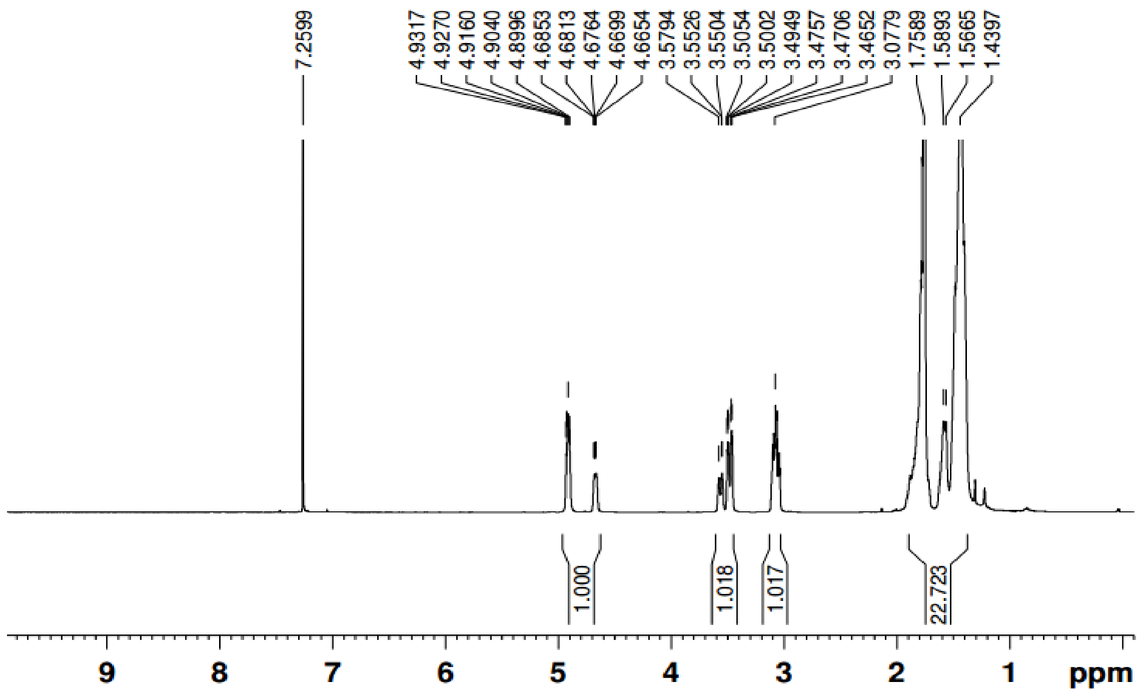

^{13}C-NMR

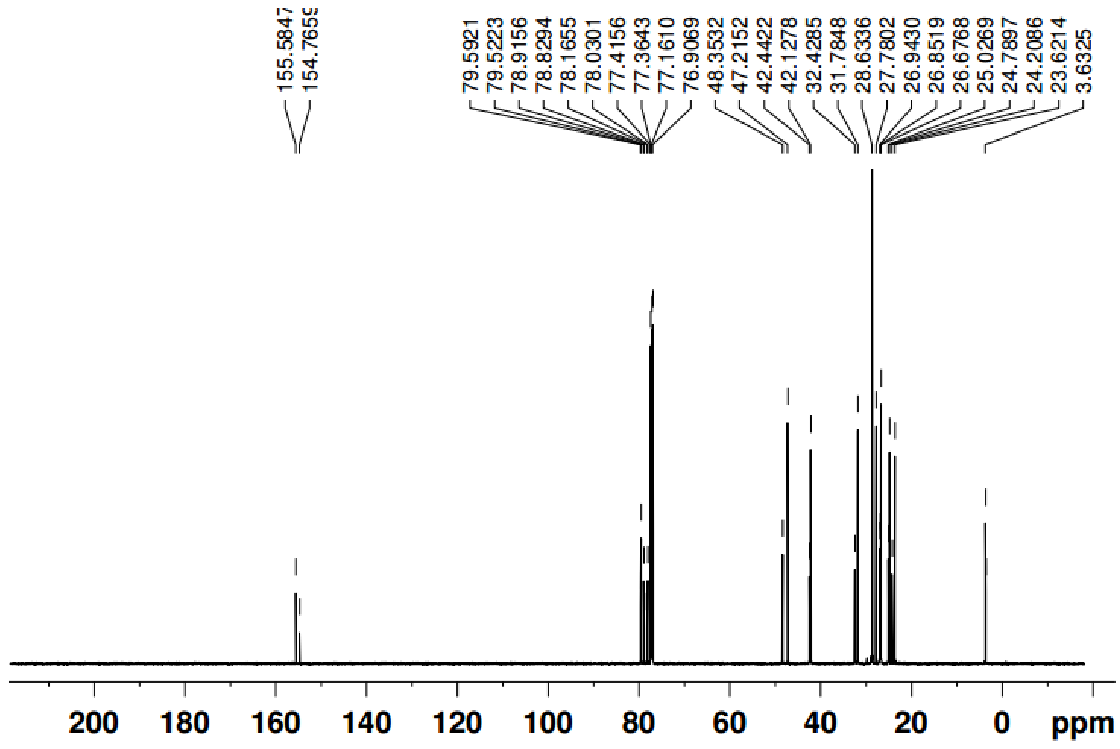

tert-Butyl 2-(prop-1-yn-1-yl)azonane-1-carboxylate (**5l**)

^{1}H-NMR

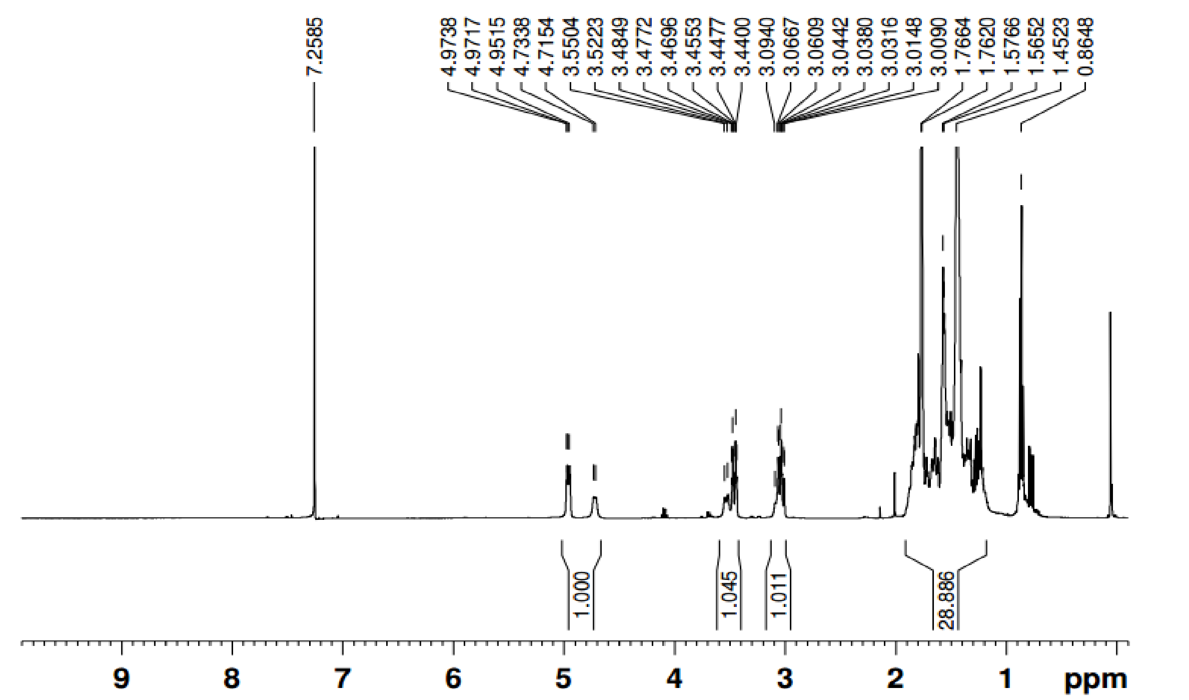

^{13}C-NMR

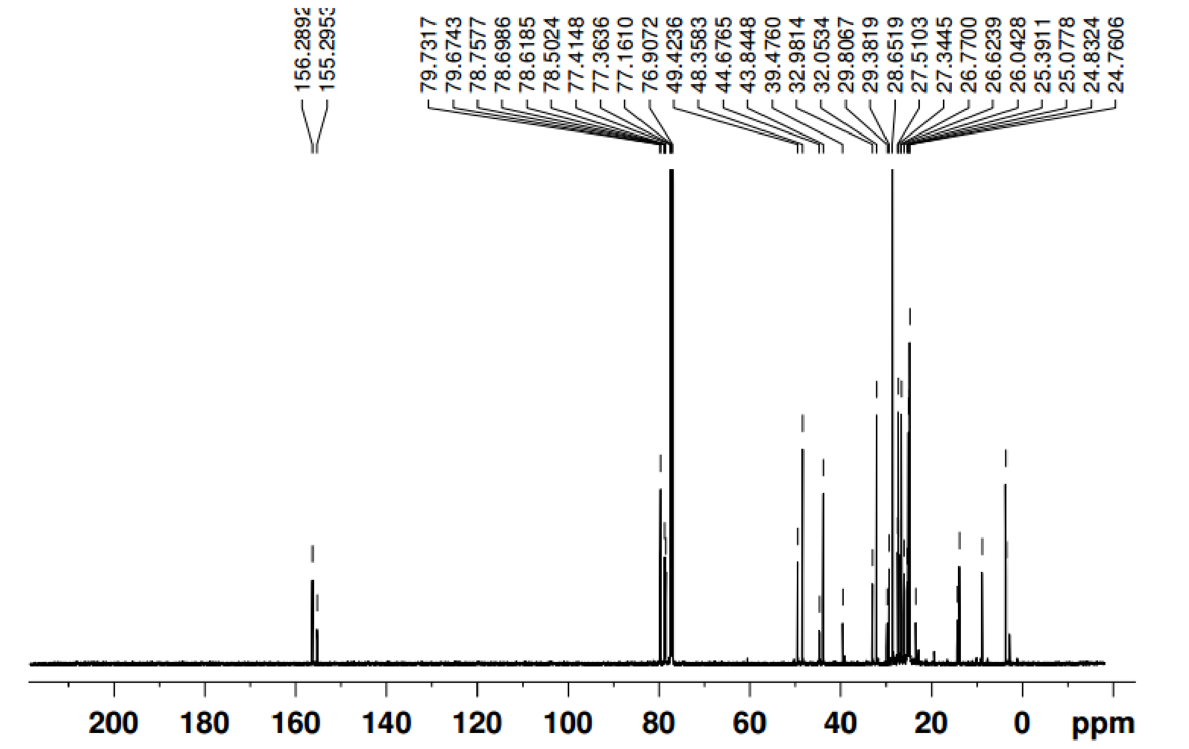

tert-Butyl 2-(phenylethynyl)piperidine-1-carboxylate (**5m**)

^{1}H-NMR

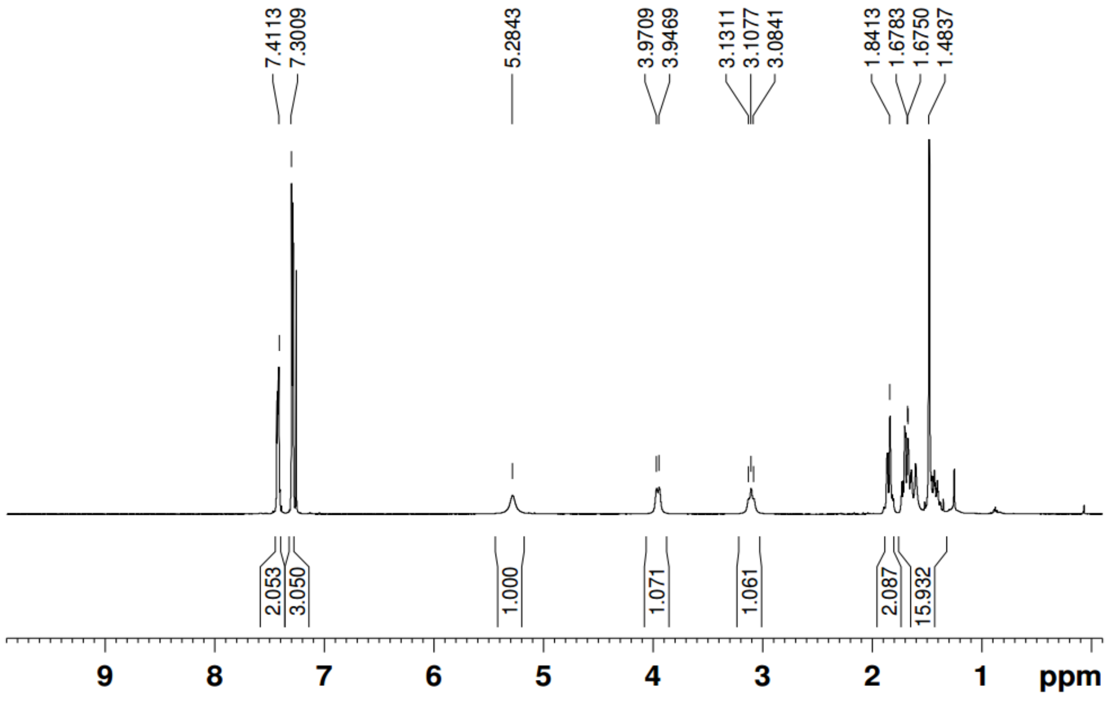

^{13}C-NMR

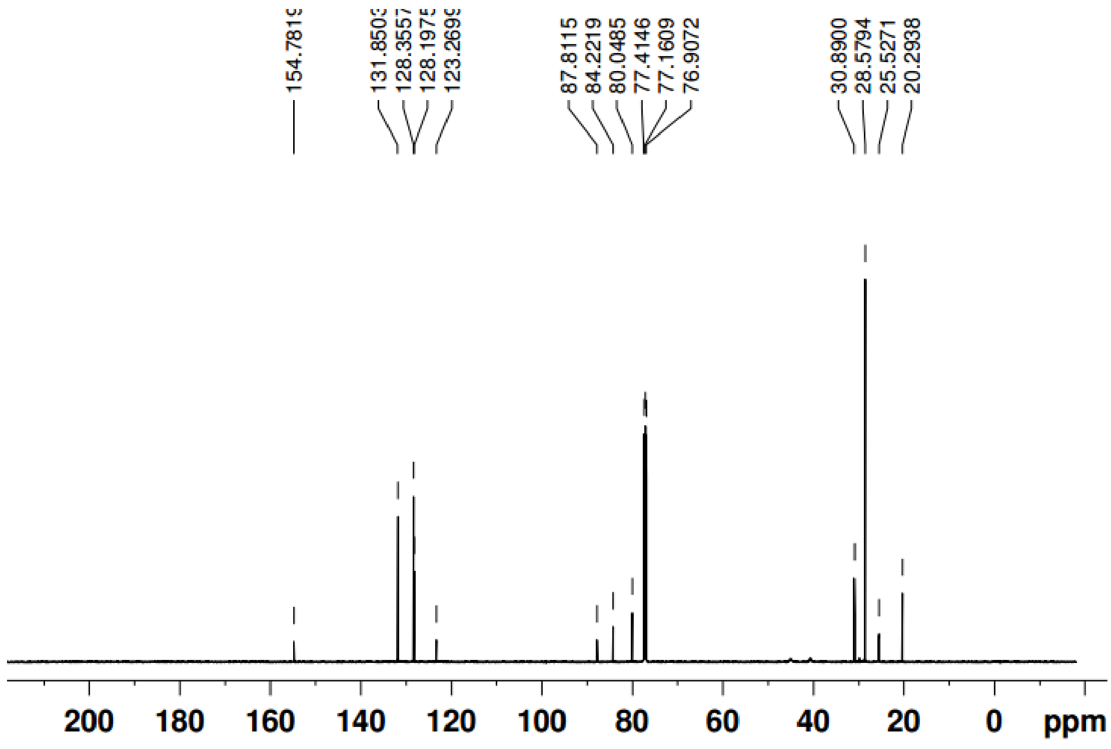

tert-Butyl 2-(phenylethynyl)azepane-1-carboxylate (**5n**)

^{1}H-NMR

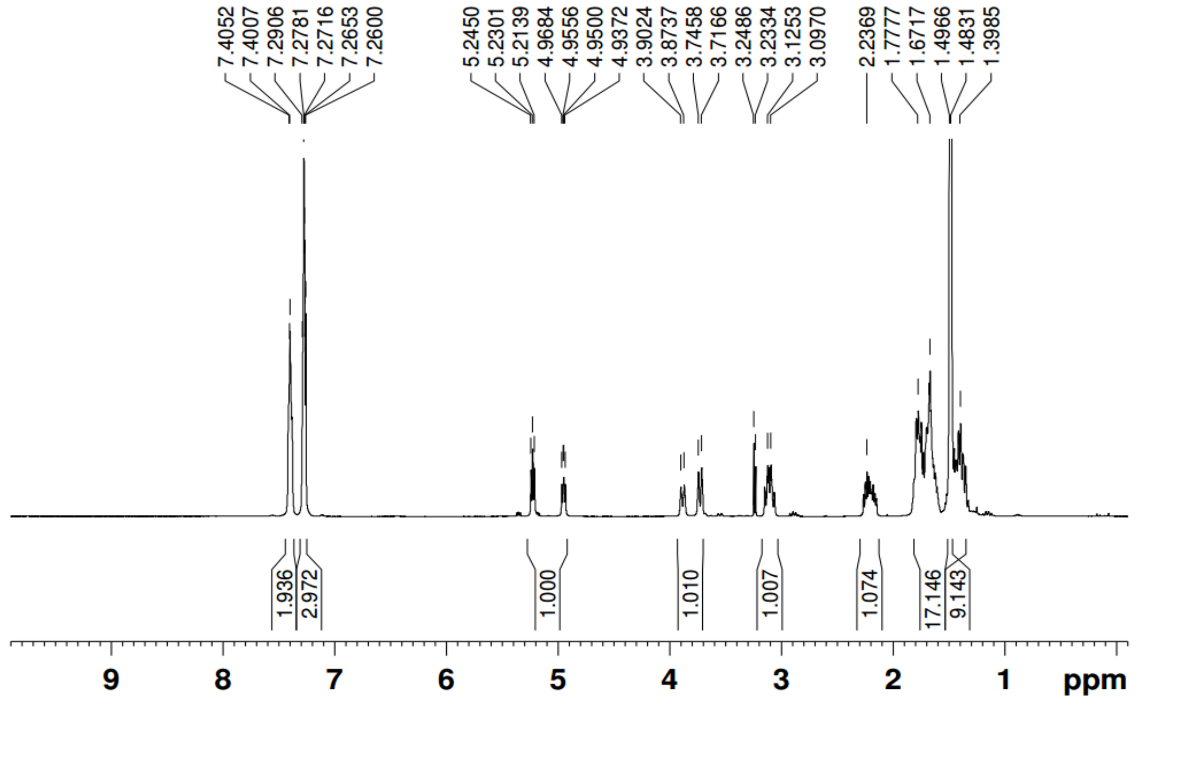

^{13}C-NMR

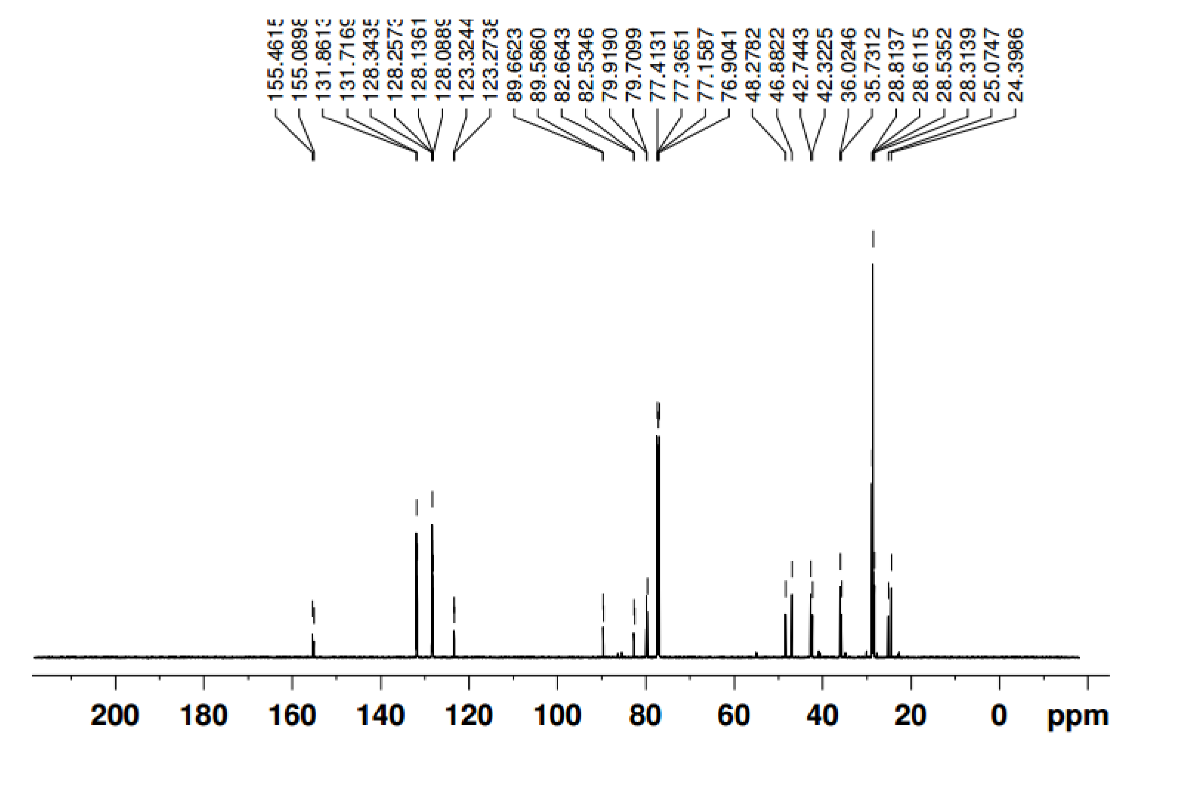

tert-Butyl 2-(phenylethynyl)azocane-1-carboxylate (**5o**)

^{1}H-NMR

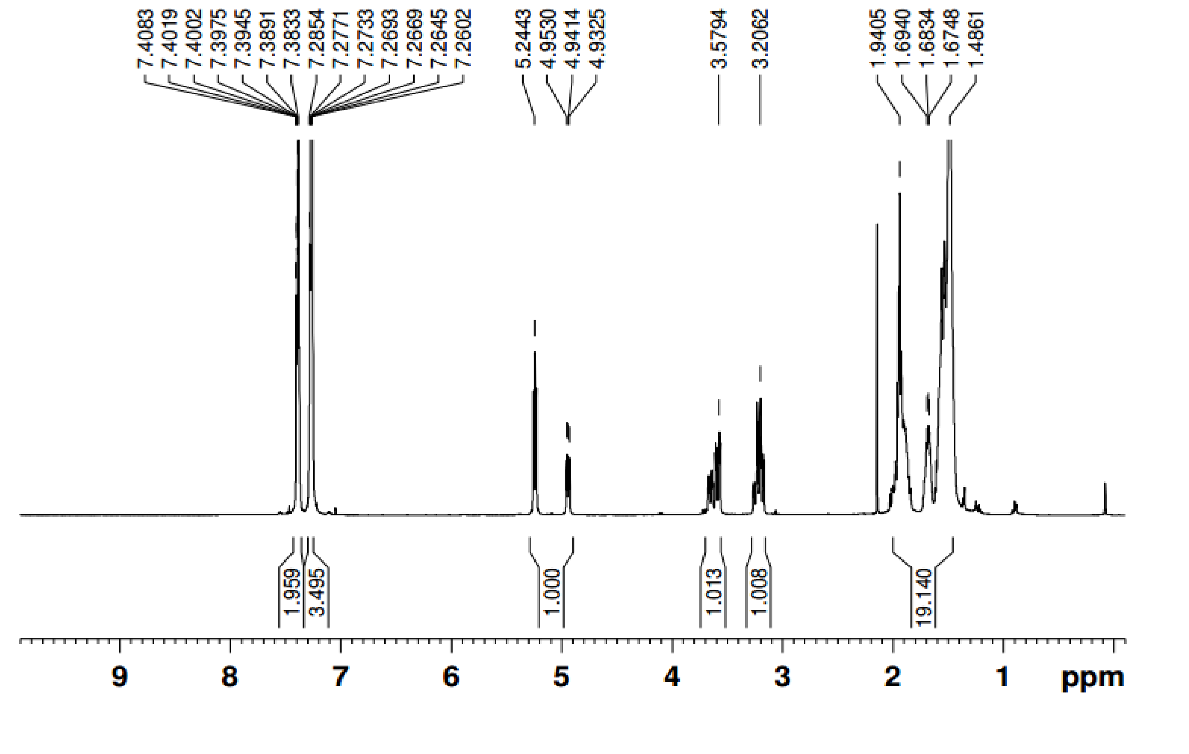

^{13}C-NMR

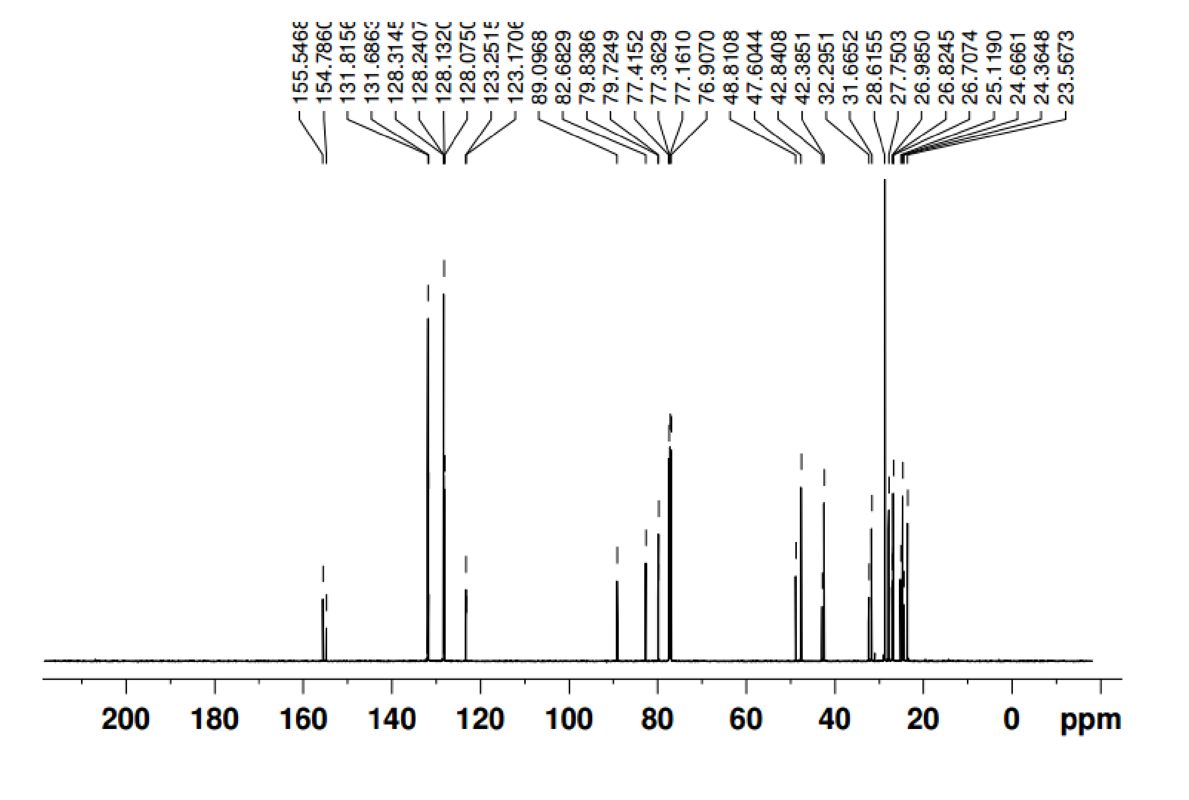

tert-Butyl 2-(phenylethynyl)azonane-1-carboxylate (**5p**)

¹H-NMR

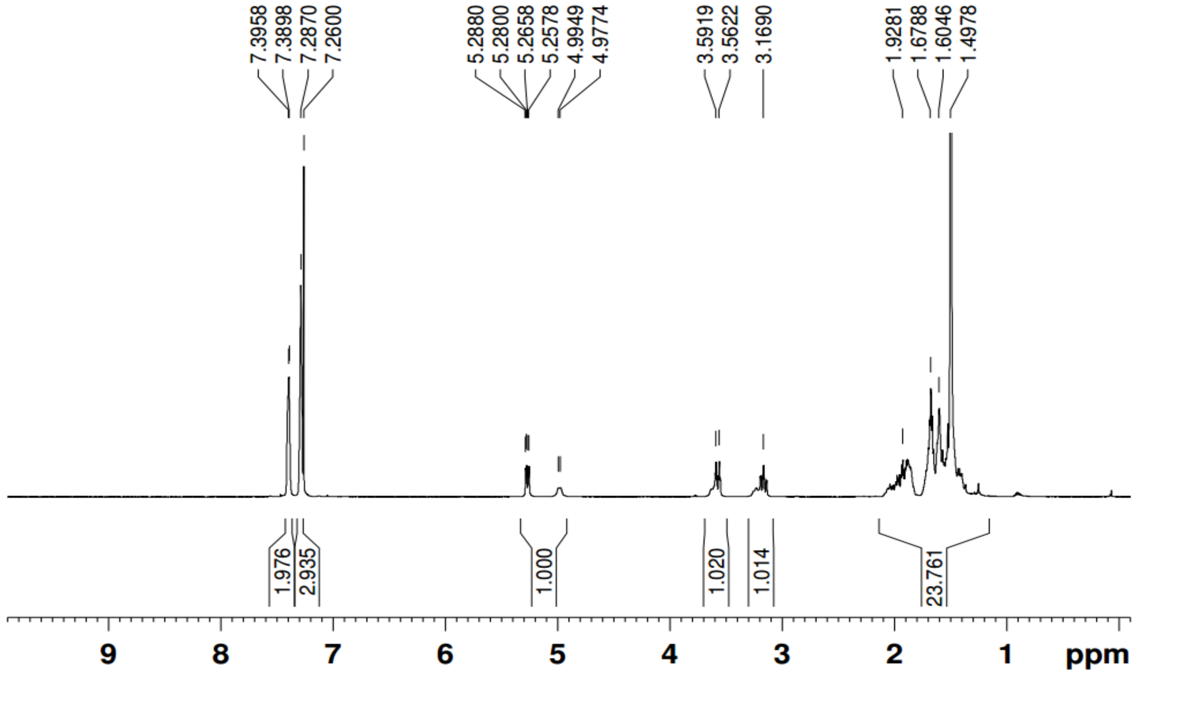

¹³C-NMR

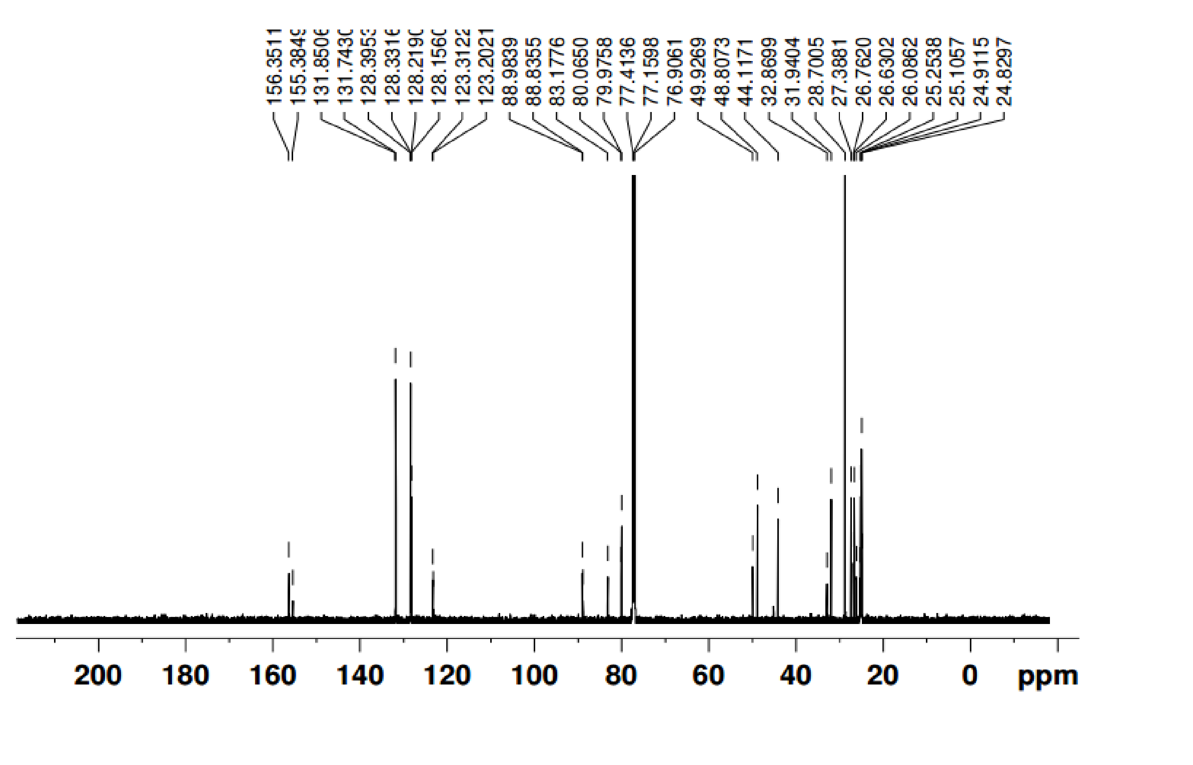

Supplement: Supplementary file 1 [file molecules-23-03023-s001.pdf]
